# Development and Validation of a High‐Throughput Screening Assay for the Legionella ADP‐Ribosyl Transferase SdeA

**DOI:** 10.1002/cbic.202500513

**Published:** 2025-09-29

**Authors:** Halana C. Vlaming, Vito Pol, Bjorn R. van Doodewaerd, Angeliki Moutsiopoulou, Paul P. Geurink, Robbert Q. Kim, Gerbrand J. van der Heden van Noort

**Affiliations:** ^1^ Leiden University Medical Centre Dept. Cell and Chemical Biology Leiden 2333 ZC The Netherlands

**Keywords:** ADP‐ribosylation, inhibitor screen, legionella, ubiquitin

## Abstract

Ubiquitination of proteins is one of the most crucial post‐translational modifications in eukaryotic cells, typically involving conjugation of ubiquitin to a lysine residue in a substrate using a three‐enzyme cascade that relies on ATP as energy source. The pathogen *Legionella pneumophila*, in contrast, employs a totally divergent pathway to ubiquitinate cellular host proteins in an unconventional manner that is crucial for bacterial proliferation. This multistep process is orchestrated by effector proteins from the SidE family which initially use NAD^+^ to adenosine diphosphate (ADP)‐ribosylate ubiquitin in a mono‐ADP‐ribosyltransferase (mART) domain. The subsequent step relies on SidE phosphodiesterase activity to conjugate phosphoribosyl‐Ubiquitin to serine residues in host substrates. Through these phosphoribosyl‐ubiquitinating events, *Legionella* is able to gain local control over the host's ubiquitin system and simultaneously evades immune responses. Hence, pursuing new inhibitors which can disrupt these crucial steps in bacterial infection are essential towards further understanding and potentially blocking *Legionella* infection. Here, we present the application of an *ε*‐NAD^+^ consumption‐based fluorogenic assay to identify small molecule modulators of the SdeA effector enzyme in a High‐Throughput Screening format, where over 600 compounds were screened. As a result, a potent inhibitor named cephalosporin C Zn^2+^ salt was discovered showing an IC_50_ of 221 nM. To investigate the inhibitory properties more deeply, various cephalosporin analogs were synthesized where variations in charge and carbon length were introduced and their inhibitory efficiencies measured and compared. Our findings suggest that the inhibition is primarily attributed to the presence of the Zn^2+^ ion, rather than the cephalosporin core. We next compared the inhibitory potential of other bivalent metal ions, illustrating that the zinc ion causes the best inhibition of the *Legionella* effector.

## Graphical abstract

We show the optimization and application of a fluorogenic assay to monitor Legionella SdeA activity in a High‐Throughput‐Screening campaign.

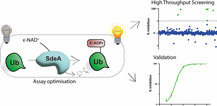

## 1 Introduction

**Figure 1:**
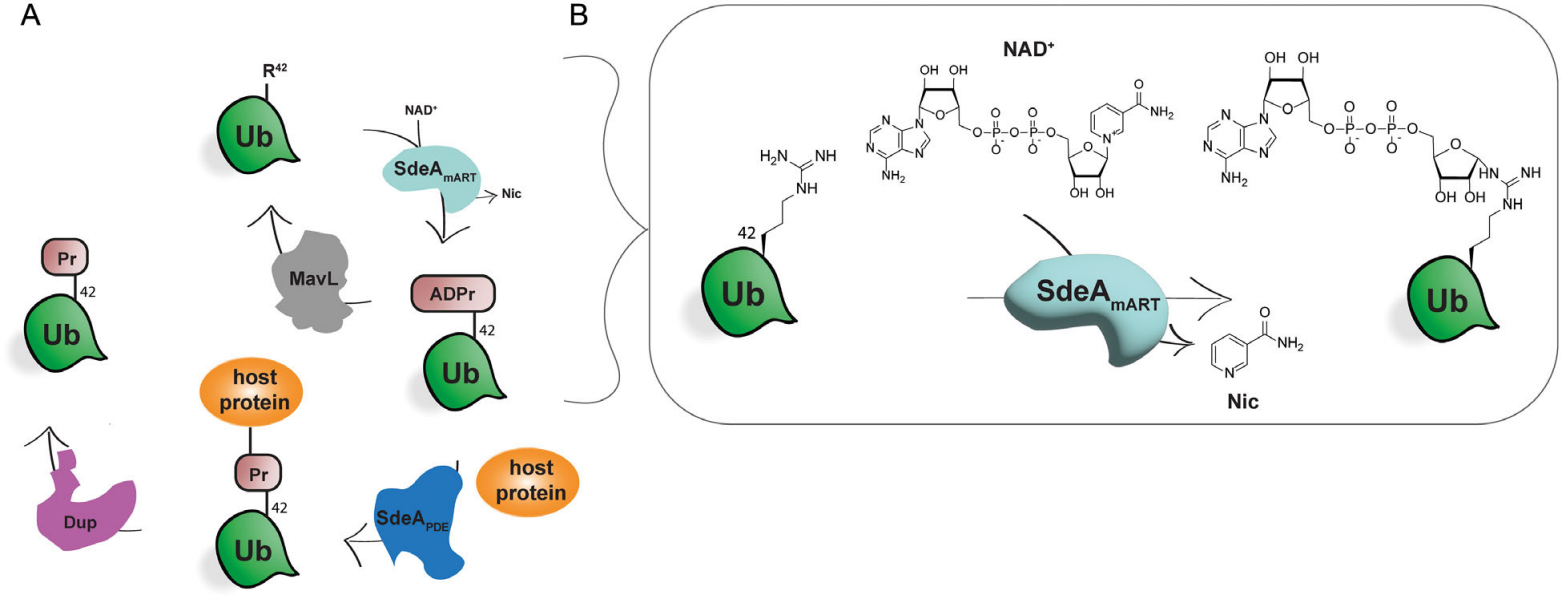
A) Schematic representation of the phosphoribosyl ubiquitination cascade used by *Legionella*. Ubiquitin (Ub) is depicted in green, the mART domain is depicted in light blue, and the PDE domain is depicted in dark blue. DupA/DupB is depicted in purple, and glycohydrolase MavL is depicted in gray. The host protein is depicted in orange. B) Zoom in on mART domain activity, where the arginine on position 42 of Ub is covalently attached to the anomeric center of the distal ribose of NAD^+^ catalyzed by SdeA. Nic: nicotinamide.

Post‐translational modifications (PTMs) are covalent attachments mostly on amino acid side chains of proteins after protein biosynthesis that play vital roles in regulation of cellular process.^[^ ^1^ ^,^ ^2^ ^]^ The PTM itself can be relatively small such as the abundant and well‐known phosphorylation event.^[^ ^3^ ^,^ ^4^ ^]^ Another frequently occurring and far larger example is ubiquitination, where a 76‐amino acid long protein is conjugated to a substrate protein. This is mediated by an E1‐E2‐E3 enzyme cascade that regulates numerous fundamental cellular processes including proteasome mediated protein degradation and DNA damage responses.^[^ ^5^, ^6^ ^–^ ^7^ ^]^ Yet, another PTM is adenosine di‐phosphate (ADP)‐ribosylation, where the attachment of ADP‐ribose to proteins via *N‐*, *O‐*, or *S‐* glycosidic linkages is catalyzed by ADP‐ribosyl transferase (ART) enzymes that consume NAD^+^.^[^ ^8^ ^,^ ^9^ ^]^ These “writers” (ARTs) are counteracted by “erasers” called ADP‐ribosylhydrolases and ‐glycohydrolases, making ADP‐ribosylation a reversible and dynamic process.^[^ ^8^ ^,^ ^10^, ^11^, ^12^ ^–^ ^13^ ^]^ On a molecular level, the conjugation of ADP‐ribose follows an SN_1_‐like mechanism in the catalytic domain of all ARTs.^[^ ^14^ ^]^ ARTs can however be divided into two subgroups, ART‐C‐ and ART‐D‐type, named after the toxins in which they were first described: *Vibrio cholerae* and *Corynebacterium diphtheria*, respectively.^[^ ^15^ ^]^ One of the differences between these classes lies in the active site, where ART‐C deploys an arginine‐serine‐glutamate (RSE) triad and ART‐D a histidine‐tyrosine‐glutamate (HYE) triad. Besides, these two groups also other motifs exist, for example in the pathogen *Chromobacterium violaceum* which during infection uses a D‐E motif, hereby blocking the ubiquitination system of the host by ADP‐ribosylating ubiquitin at Thr66.^[^ ^16^ ^,^ ^17^ ^]^ Another pathogen which also operates by ADP‐ribosylation of ubiquitin during infection is the bacterium *Legionella pneumophila*, where it uses multiple ADP‐ribosylation effectors resulting in ADP‐ribosylation of Arg42 of ubiquitin.^[^ ^18^ ^,^ ^19^ ^]^ *Legionella* is a gram‐negative bacterium that thrives in fresh water environments or moist soil and uses a host to procreate.^[^ ^20^ ^,^ ^21^ ^]^ Humans can be infected via inhalation of contaminated aerosols, during which the bacterium enters the host cell. In the cytosol, *Legionella* creates a *Legionella*‐containing vacuole (LCV) to escape detection and destruction by intracellular defense mechanisms.^[^ ^22^ ^]^ *Legionella* uses a multitude of effector proteins to build and maintain the LCV, thereby manipulating intracellular processes like protein translation, vesicle trafficking, and gene expression.^[^ ^23^, ^24^, ^25^ ^–^ ^26^ ^]^ A group of these effector proteins called the SidE family, consisting of SidE, SdeA, SdeB and SdeC, plays an important role by controlling LCV integrity and vesicle transport in an unconventional manner. These multidomain proteins use their mART domain (SdeA_mART_, **Figure** **1**) to ADP‐ribosylate host ubiquitin on Arg42. Next, the phosphodiesterase (PDE) domain (SdeA_PDE_, Figure 1) catalyzes attachment of a serine containing host cell substrate protein via a phosphodiester bond to the ADP‐ribosylated ubiquitin, expelling adenosine‐monophosphate in the process, while forming a phosphoribosyl linkage between ubiquitin and the host substrate.^[^ ^23^ ^,^ ^27^ ^,^ ^28^ ^]^ Acting in total contrast to canonical ubiquitination, *Legionella* evades the need for utilizing ATP or E1, E2 and E3 enzymes, resulting in controlling the ubiquitination of proteins involved in predominantly ER and Golgi systems.^[^ ^29^ ^–^ ^31^ ^]^ It has been shown that deletion of these SidE effectors results in a reduction of bacterial growth, emphasizing the importance of the SidE family for *Legionella* reproduction.^[^ ^32^ ^]^ Formed phosphoribosyl (Pr) ubiquitination of host substrates a.o. interrupts GTP‐loading and hydrolytic activity of Rab‐GTPases, in addition to rearrangement and fragmentation of the ER.^[^ ^23^ ^,^ ^30^ ^,^ ^33^ ^]^ To retain dynamic control, *Legionella* counteracts these SidE ligating activities via deubiquitinases DupA and DupB, by deconjugating the Ub^Pr^ from the serine substrate, as tight regulation of phosphoribosylated ubiquitin is crucial for the survival of the host cell, hence also for *Legionella* itself.^[^ ^27^ ^,^ ^30^ ^]^ Subsequently, Ub^Pr^ can be converted back to ADP‐ribosylated ubiquitin by effector protein LnaB, an AMPylase dependent on co‐factor actin,^[^ ^34^, ^35^, ^36^ ^–^ ^37^ ^]^ or conversily *Legionella* effector MavL, a glycohydrolase, can reverse the ADP‐ribosylation of ubiquitin. Glutamylase SidJ also helps to control the phosphoribosyl ubiquitination status by oppressing SdeA activity in the first step of the cascade.^[^ ^38^, ^39^, ^40^, ^41^, ^42^ ^–^ ^43^ ^]^ Pr‐ubiquitination of proteins and the preceding ADP‐ribosylation of Ub has not been shown to be effected by mammalian enzyme activities. Due to its uniqueness in *Legionella* infection and the fact that SidE proteins are crucial for *Legionella* proliferation, inhibiting this ART function may hence contribute to further clinical applications as future therapeutic drugs might reduce bacterial proliferation or reduce bacterial immune evasion. We therefore present a High‐Throughput Screening campaign based on a fluorogenic assay using *ε*‐NAD, resulting in a fluorescent signal upon SdeA activity. Approximately 600 small molecules were screened, resulting in the identification of the zinc salt of cephalosporin C as apparent hit, showing an IC_50_ of 0.2 μM. Subsequently a small library of cephalosporin analogs was synthesized to gain more information on their inhibiting effects.

## 2 Results and Discussion

### 2.1 Development of a Fluorogenic Assay to Monitor SdeA Activity

**Figure 2:**
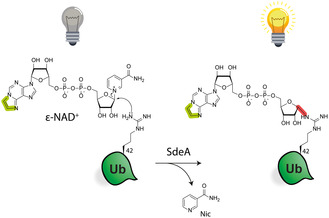
ADP‐ribosylation of ubiquitin with *ε*‐NAD catalyzed by SdeA. The etheno bridge is marked in green. The formed covalent bond is marked in red.

We deemed a fluorogenic assay based on the consumption of *N* ^1^,*N* ^*6*^‐ethenoadenine dinucleotide (*ε*‐NAD^+^)(44) to monitor SdeA activity as a good starting point as it was previously successfully applied by us and others.^[^ ^44^ ^,^ ^45^ ^]^ In contrast to regular NAD^+^, *ε*‐NAD contains an etheno bridge in the nucleobase between primary amine *N* ^1^ and secondary amine *N* ^6^ in the adenine ring (**Figure** **2**). Due to this element, the nucleobase acts as a fluorescent dye upon excitation, but its fluorescence however is intramolecularly quenched by the nicotinamide group in the *ε*‐NAD^+^ dinucleotide stage. *ε*‐NAD^+^ itself is thus not fluorescent, but when the arginine on position 42 of ubiquitin (Ub) performs a nucleophilic attack on the anomeric center of ribose of *ε*‐NAD^+^ catalyzed by SdeA, the nicotinamide group is detached and quenching is abrogated. As such, not only a novel covalent bond between the adenosine diphosphate ribose part and ubiquitin is formed (highlighted in red, Figure2), but the product of the reaction becomes fluorescent upon excitation with light at a wavelength of 300 nm.

**Figure 3:**
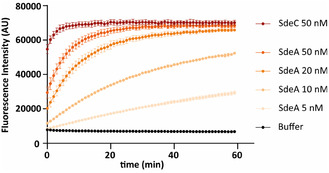
ADP‐ribosylation of Ub with *ε*‐NAD^+^ catalyzed by SdeA (5–50 nM), demonstrated by increase of the fluorescence intensity over a period of 1 h. Substrate used: *ε*‐NAD^+^/Ub (1:1): 50 μM. The measurement was conducted in triplicates, data are presented as mean values +/− SEM.

*ε*‐NAD^+^ was synthesized by treating regular NAD^+^ with chloroacetaldehyde in an acidic environment, as previously described.^[^ ^46^ ^]^ Full‐length Ub_1–76_ was synthesized by SPPS^[^ ^47^ ^]^ or expressed and purified by HPLC and gel‐filtration. Recombinantly expressed SdeA was added to a 1:1 ratio of 50 μM Ub and *ε*‐NAD^+^ substrates,^[^ ^44^ ^]^ and fluorescence intensity (FI) was recorded for 1 h (*λ*_ex/em_ = 320/380 nm) at room temperature. Various concentrations of SdeA were investigated to maximize the assay window (**Figure** **3**). Previously applied SdeC was used here as a positive control,^[^ ^44^ ^]^ and buffer only was used as negative control.

At enzyme concentrations of 50 nM and 20 nM, the turnover rate is substantially higher than at the lower concentrations, resulting in the plateau of the curve being reached in the first 20 min. At 5 nM, a linear progress is observed but with a rather slow increase in FI compared to 10 nM. Therefore, 10 nM was chosen as optimal enzyme concentration for the assay, showing a near‐linear increase over the assayed 60 min.

### 2.2 High‐Throughput Screening Campaign

After assessing SdeA activity, our next objective was to determine whether the assay could be adapted into a High‐Throughput Screening format to find inhibitors in an efficient manner. As the desired effect is the inhibition of SdeA by a druglike compound, we tested several small molecules as potential positive controls, (3‐aminobenzamide (3‐AB), *N*‐succinimidyl octanoate, and phenylmethylsulfonyl fluoride (PMSF)), to see if any of these compounds would produce a similar effect and hence can serve as a positive control. 3‐Aminobenzamide (3‐AB) is a known PARP inhibitor^[^ ^48^ ^]^ but appeared to be auto‐fluorescent. As SdeA contains an R‐S‐E catalytic triad, serine inhibitor phenylmethylsulfonyl fluoride (PMSF) was tested^[^ ^49^ ^]^ along with *N*‐succinimidyl octanoate which has been reported to react with serine residues in addition to lysine and cysteine.^[^ ^50^ ^]^ The latter showed full inhibition at a concentration of 1.25 mM with a preincubation time of 30 min at room temperature and was therefore selected as positive control (100% inhibition), and DMSO (1% v/v) was selected as negative control (0% inhibition) (Figure S1, Supporting Information). To determine the quality of the assay to be suitable for HTS, a Z′ value was calculated. The Z′ value is a crucial statistical measure reflecting the reliability of the assay, where a Z′ > 0.5 is seen as excellent assay quality.^[^ ^51^ ^]^ To select an optimal HTS timepoint with a good Z′ value together with an adequate assay window, the assay was executed using 100 replicates of positive controls and negative using 10 nM of SdeA and 50 μM of *ε*‐NAD^+^/Ub (1:1) and an incubation time of 30 min at room temperature. Next, fluorescence intensity (FI) was recorded for one hour (Figure S2, Supporting Information). We chose the 28‐minute timepoint because of a proper Z′ valuable in combination with a decent assay window, while remaining within the linear stage of the enzymatic reaction.

**Figure 4:**
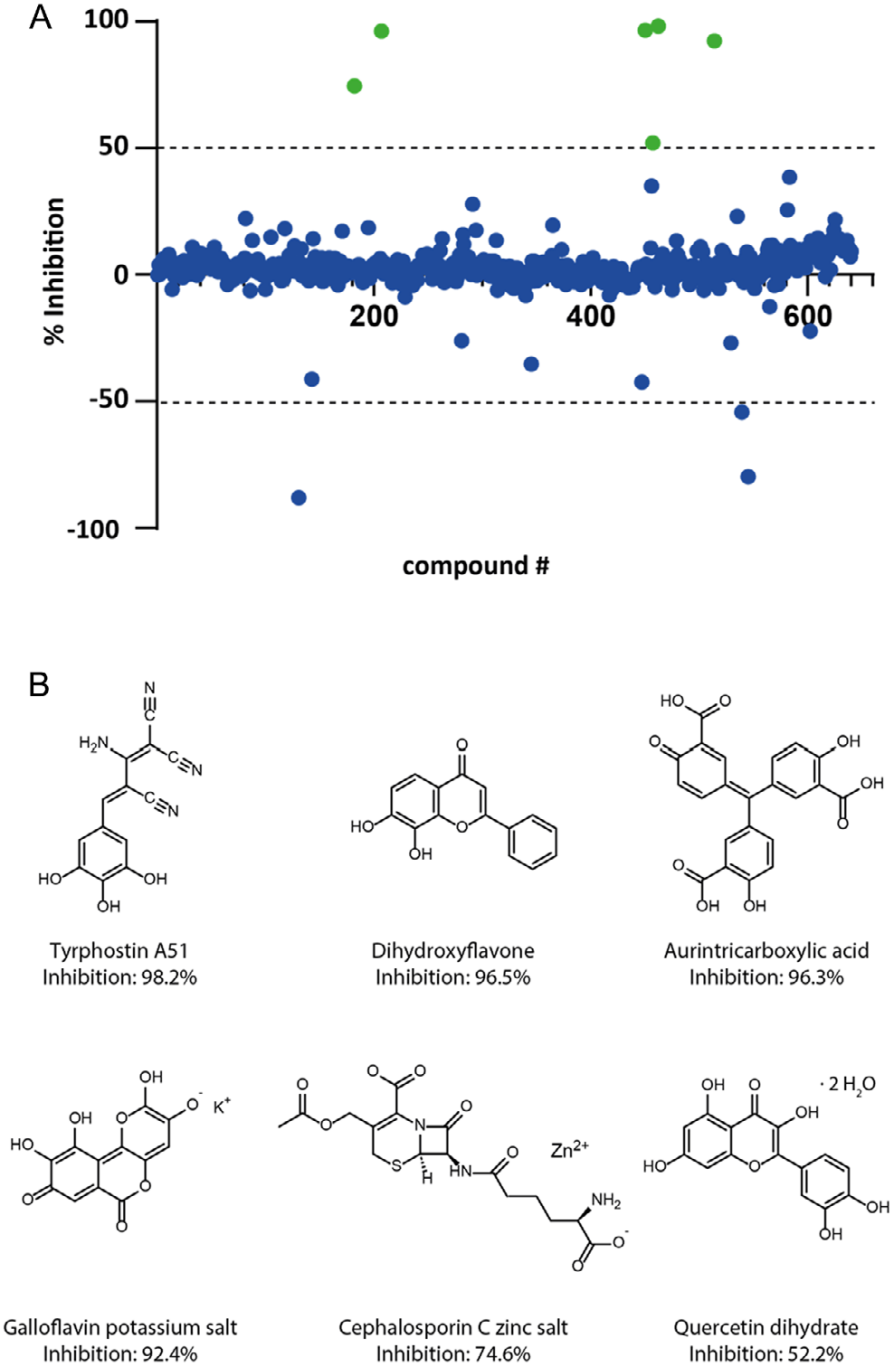
A) Results of the High‐Throughput Screening of the LOPAC library, where the relevant hits are indicated in green. B) Structures of the six hits with inhibition levels (%) at a concentration of 5 μM.

We subsequently screened the widely used “Library of Pharmacologically Active Compounds” (LOPAC) known for its versatile collection of pharmacologically relevant compounds, entailing marketed drugs or clinical candidates.^[^ ^52^ ^]^ Six hundred forty compounds were screened at a final compound concentration of 5 μM in a 384‐well format with a total volume of 20 μL per well. The percentage inhibition of each compound was calculated from the measured FI values after normalization to the positive (*N*‐succinimidyl octanoate, 100% inhibition) and negative (DMSO, 0% inhibition) controls. For this screen, the average Z′ value over two plates was determined to be 0.92, verifying the reliability of this approach. As a result, six compounds out of the 640 screened compounds showed inhibition >50% (**Figure** **4**) and were studied further.

**Figure 5:**
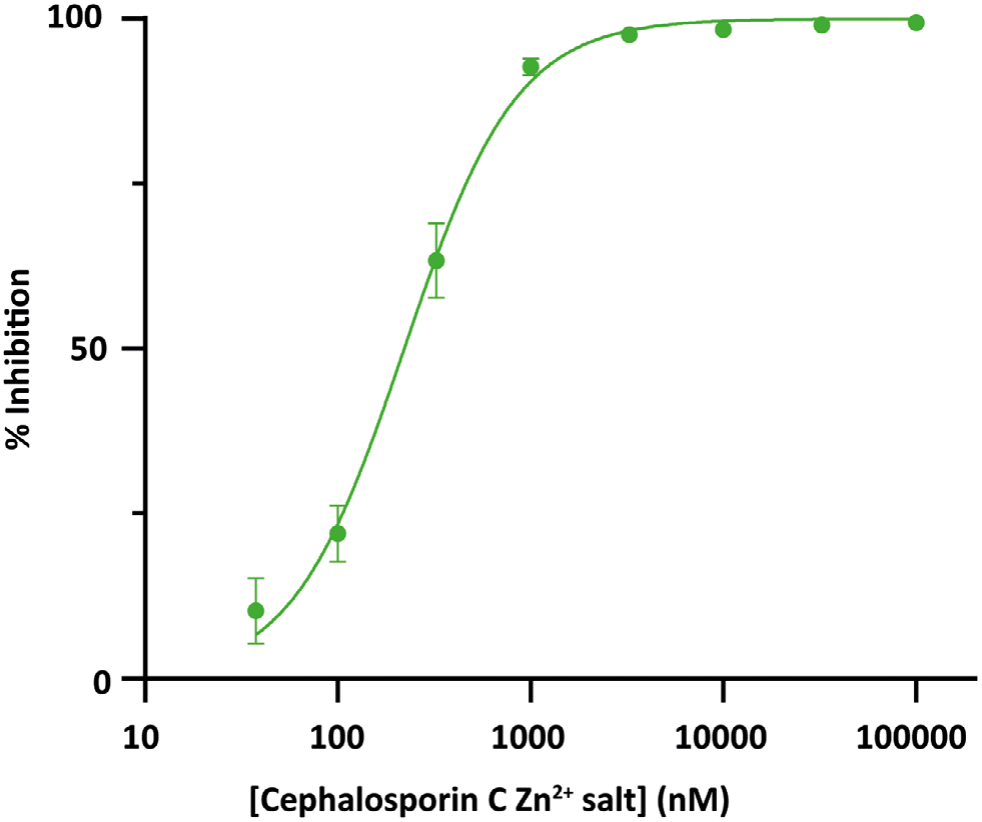
IC_50_ determination of potent hit cephalosporin C Zn^2+^ salt with an IC_50_ of 221 nM. The measurement was conducted in technical triplicates, data are presented as mean values +/− SEM.

Next, a Pan‐Assay Interference Compounds (PAINS)^[^ ^53^ ^]^ filter was applied to eliminate false positives and frequent hitters when screening a library.^[^ ^54^ ^]^ Tyrphostin A51, dihydroxyflavone, galloflavin, and quercetin dihydrate were all flagged as PAINS because of catechol groups, while aurintricarboxylic acid could interfere with absorption of light. Only one compound survived the PAINS filter which was the zinc salt of cephalosporin C, showing an inhibition of 75% at 5 μM. To obtain an estimate about the inhibition efficiency of the hit, a concentration series of cephalosporin C Zn^2+^ salt was incubated with SdeA (10 nM final concentration) for 30 min, followed by addition of substrate *ε*‐NAD^+^/Ub (50 μM final concentration, 1:1 ratio). The FI was measured for 1 h, and the subsequent values were normalized to the positive control (*N*‐succinimidyl octanoate, 100% inhibition) and negative control (DMSO, 0% inhibition) to calculate the inhibition percentage. These inhibition values were plotted against the concentration of cephalosporin C Zn^2+^ salt, from which an IC_50_ of 221 nM was calculated (**Figure** **5**).

### 2.3 Synthesis and Evaluation of Cephalosporin C Analogs

**Figure 6:**
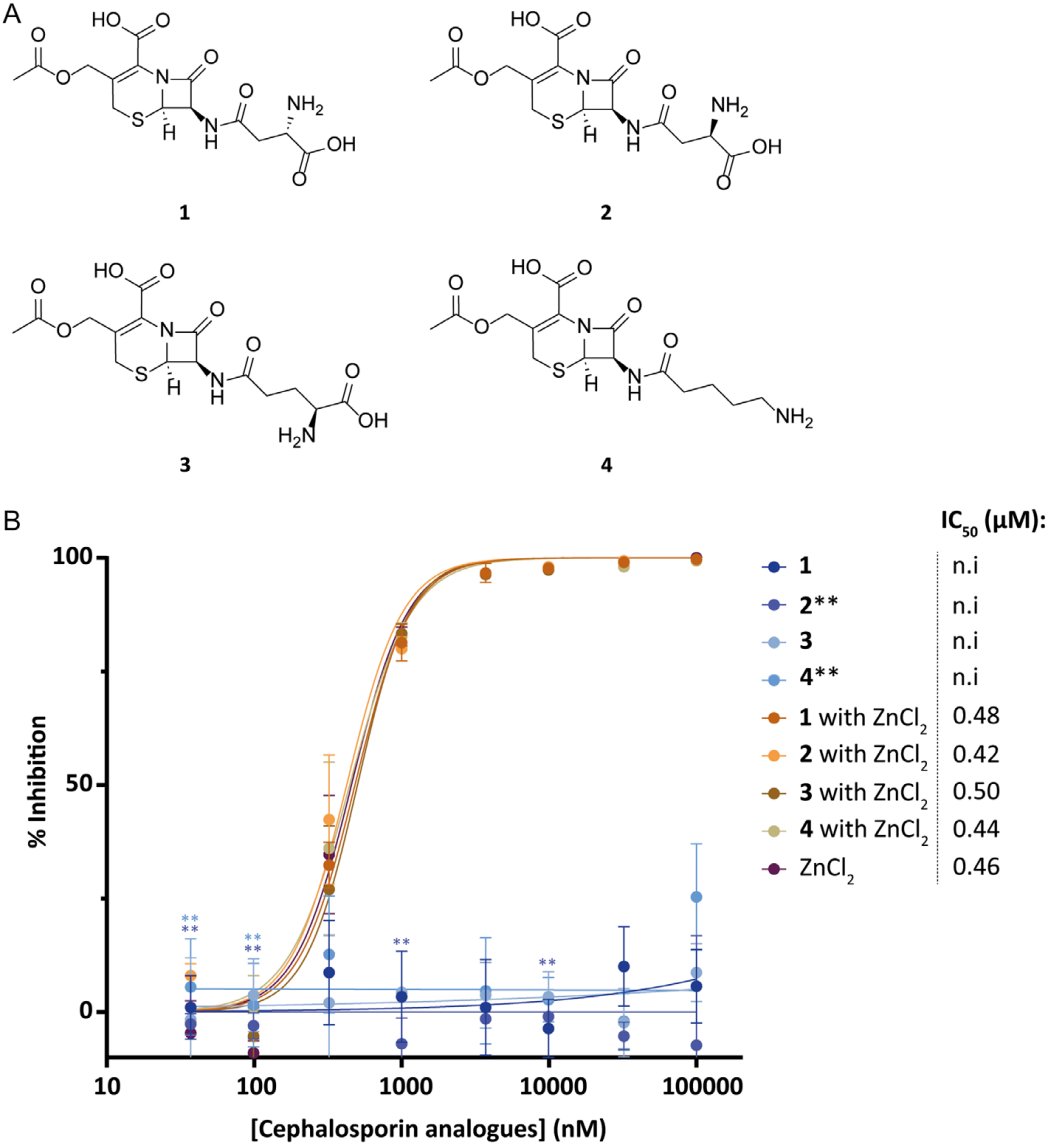
A) Cephalosporin analogs **1–4** synthesized from 7‐aminocephalosporic acid. B) IC_50_ determination of cephalosporin analogs asparagine **1–4** without ZnCl_2_ added (blue colors) and with ZnCl_2_ added (brownish colors). ZnCl_2_ (purple) was taken along as control. The measurement was conducted in triplicates, data are presented as mean values +/− SEM.**: datapoints were based on duplicates only. n.i: no inhibitory effect.

Cephalosporins are one of the four classes (penicillin, carbapenem, monobactam, and cephalosporin) of *β*‐lactam antibiotics.^[^ ^55^ ^]^ *β*‐Lactam antibiotics are one of the most successful antibiotics and widely used to treat both gram‐positive and gram‐negative bacteria.^[^ ^55^ ^,^ ^56^ ^]^ Their mechanism of action typically relies on inhibition of cell wall synthesis, capturing the peptidase activity of the penicilin‐binding protein.^[^ ^57^ ^]^ As also other cephalosporins were included in the screened LOPAC library but did not show inhibition in our assay, we opted to investigate the source of the observed inhibition from this particular cephalosporin C. This hit contained a D‐amino adipic acid conjugated to the 7‐aminocephalosporanic acid core. We therefore constructed several cephalosporin analogs differing in this adipic acid group, varying both in chain length and charge (**Figure** **6**A) to investigate the effect on SdeA inhibition. The different moieties were attached to the *β*‐lactam core (7‐aminocephalosporanic acid, scheme S1, Supporting Information) by first protecting the free carboxylic acid with a tert‐butyl group, followed by attaching protected D‐asparagine, L‐asparagine, D‐glutamine, and D‐amino‐valeric amino acid using standard peptide coupling reagents. Next, all protecting groups were removed under acidic conditions resulting in compounds **1**, **2**, **3**, and **4**. In addition to measuring the inhibitory efficiency of the obtained compounds (as TFA salts) alone, a combination of compounds **1**‐**4** with addition of ZnCl_2_ were tested, making sure a fair comparison would be made with the original cephalosporin C hit Zn^2+^ salt (Figure 6B).

**Figure 7:**
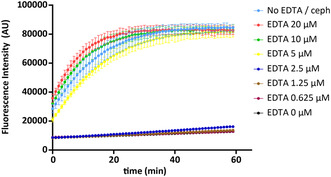
SdeA activity at different EDTA concentrations with a fixed concentration (5 μM) of cephalosporin C Zn^2+^ salt. The light colors blue, red, green and yellow depict when [EDTA] > [cephalosporin], and the dark colors blue, brown, purple and black depict when [EDTA] < [cephalosporin]. The measurement was conducted in triplicates, data are presented as mean values +/− SEM.

We observed that compounds **1–4** by themselves displayed no inhibitory effect, while once supplied with Zn^2+^ did show inhibition. Notably the ZnCl_2_ only control showed a similar inhibition. These results indicated that the Zn^2+^ ion plays a role in the inhibition of SdeA mediated ADP‐ribosylation of ubiquitin. To validate our hypothesis, we conducted an experiment where hit cephalosporin C Zn^2+^ salt was incubated with various concentrations of EDTA (**Figure** **7**), a known metal ion chelator,^[^ ^58^ ^]^ prior to SdeA addition (10 nM final concentration). After incubating for 30 min at room temperature, substrate *ε*‐NAD^**+**^/Ub (50 μM final concentration, 1:1 ratio) was added, and the fluorescence intensity was measured for 1 h. The experiment revealed that when the concentration of EDTA exceeds the concentration of cephalosporin C Zn^2+^ salt, the enzymatic activity of SdeA is retained. In contrast, when the concentration of EDTA is lower than the amount of cephalosporin C Zn^2+^ salt, the inhibition of the enzyme was observed as almost no increase in fluorescent signal is detected. This strongly suggests that when Zn^2+^ is sufficiently chelated by EDTA, it is unable to inhibit the enzyme, but if EDTA is unable to chelate all zinc ions, Zn^2+^ inhibits SdeA activity leading to no ADP‐ribosylation of ubiquitin and hence no increase in fluorescent signal.

Zn^2+^ ‐ions have been shown to inhibit other enzymes. It has been reported that Zn^2+^ acts as an inhibitor of proton transfer for several enzymes ranging from photosynthetic reactions to transhydrogenases and can also inhibit allosterically as is shown for caspase‐6 and −9.^[^ ^59^, ^61^, ^62^ ^–^ ^63^ ^]^ Furthermore, multiple articles state that next to Zn^2+^, also other d‐metals like Cd^2+^, Pb^2+^, Co^2+^, or Ni^2+^ can inhibit in a similar manner.^[^ ^60^ ^,^ ^64^ ^]^

**Figure 8:**
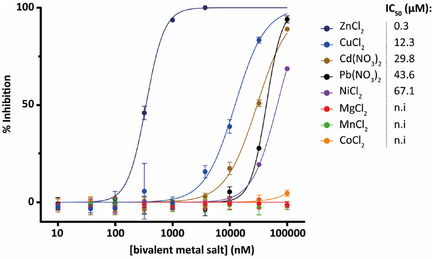
IC_50_ determination of bivalent metal ions CuCl_2_, Cd(NO_3_)_2_, Pb(NO_3_)_2_, NiCl_2_, MgCl_2_, MnCl_2_, and CoCl_2_ with their corresponding IC_50_ values depicted in μM on the right. The measurement was conducted in triplicates, data are presented as mean values +/− SEM. n.i: no inhibitory effect.

We hence tested several bivalent metal ions and determined their inhibitory potential (**Figure** **8**) on SdeA. The resulting IC_50_ values indicate a top three of Zn^2+^ > Cu^2+^ > Cd^2+^ with values 0.3 μM, 12.3 μM, and 29.8 μM respectively, showing a remarkable 40‐fold difference between zinc and copper. Meanwhile, Mg^2+^, Mn^2+^, and Co^2+^ do not show any inhibitory effect.

This suggests that SdeA inhibition in this assay is not induced by any bivalent metal but rather requires Zn^2+^ specifically to disrupt the ADP‐ribosylating activity of SdeA. Additionally, it has been reported previously that Zn^2+^ can coordinate to histidine, aspartate, or glutamate.^[^ ^65^, ^66^ ^–^ ^67^ ^]^ As the catalytic triad in the active site of SdeA in the mART domain contains a glutamate, we speculate that Zn^2+^ coordinates to this and thereby disturbs the catalytic activity. It remains the question why especially Zn^2+^ demonstrates a larger effect than for instance Cd^2+^, as this metal ion can also bind these specific residues.^[^ ^60^ ^]^ Cd^2+^ however is much larger than Zn^2+^ and therefore may have trouble fitting in the catalytic pocket, or the difference in coordination may play a role as Cd^2+^ more often complexes in octahedral form unlike Zn^2+^ which rather assumes a tetrahedral complex.^[^ ^64^ ^,^ ^67^ ^]^ Copper on the other hand, placed next to zinc in the periodic table, is about equal in size. This may lead to a better fit compared to Cd^2+^; however, still a large difference remains in inhibitory efficiencies of Cu^2+^ versus Zn^2+^. We deemed it unlikely that inhibition resulted from Zn^2+^ coordination to the substrate, as only 5 μM of Zn^2+^ salt already resulted in full inhibition compared to 50 μM substrate used in the assay.

## 3 Conclusion

*Legionella pneumophila* is a gram‐negative pathogenic bacterium thriving in aqueous environments that is able to infect humans via contaminated aerosols. It proliferates in human cells by forming a LCV where it deploys effector proteins to among others sustain this LCV via interfering and hijacking numerous processes in the cell. One of these effector proteins called SdeA does so by ADP‐ribosylating ubiquitin using NAD^+^ using its mART domain catalytic activity, leading to phosphoribosyl ubiquitination of substrate proteins, an unconventional manner of ubiquitination that as such deregulates the ubiquitin machinery and signaling within the host cell. Further elucidation of this mechanism through identifying novel inhibitors can provide deeper insights into *Legionella* infection and may lead to new clinical intervention strategies thereby potentially leading to alternative treatment for Legionnaires disease. We here present a fluorogenic assay which we optimized by determining the optimal enzyme concentration and assessing various small molecules to select the most suitable controls. We continued with a High‐Throughput Screening campaign where more than 600 small molecules were screened for their inhibitory properties, obtaining a Z′ ≈ 0.92 demonstrating the robustness of our assay. This resulted in one validated hit named cephalosporin C Zn^2+^ salt showing a potent IC_50_ value of 0.2 μM. To investigate its inhibition mechanism more deeply, a small study was conducted where cephalosporin analogs **1**‐**4** were synthesized, differing in linker length and charge in the D‐amino adipic acid part of the molecule. These cephalosporin C analogs were assessed in isolation or in presence of ZnCl_2_ for their inhibitory activity. This experiment revealed that only the compounds with a Zn^2+^ ion involved demonstrated an inhibitory effect, similar in magnitude as Zn^2+^ itself. To verify the observation that Zn^2+^ would be responsible for inhibition, cephalosporin C Zn^2+^ salt was incubated with zinc chelator EDTA prior to measuring enzymatic activity with the assay. The results showed a lack of inhibition if sufficient EDTA was present to chelate al Zn^2+^ ions, and hence enzymatic activity was maintained. Conversely, if the amount of EDTA was too low for total chelation of Zn^2+^ ions, inhibition occurred. These findings strongly suggest that Zn^2+^ itself is responsible for inhibiting the enzyme SdeA in our assay. We were curious to find out whether specifically Zn^2+^ inhibits SdeA or if any random bivalent metal ion would produce a similar effect and hence tested several bivalent metals ions for their inhibitory efficiencies. Zn^2+^ exhibited a larger inhibitory effect by a forty‐fold difference compared to other bivalent metals Cd^2+^, Pb^2+^, or Cu^2+^. Although it would be interesting to pursue this matter further and determine why and how specifically Zn^2+^ inhibits SdeA, this study marks a warning for other screening assays, as the potential (false positive) inhibitory effect of Zn^2+^ should be watched carefully. For optimizing the used protocol, metal chelators like EDTA or TPEN could be used immediately when detecting metal ions sensitive hits, as this avoids precious time, material, and effort when potentially dealing with a false positive.^[^ ^68^ ^]^ Nonetheless, the assay demonstrated here opens up the potential to screen other libraries and continue the search for inhibitors for this enzyme family involved in a so‐far bacterium‐specific and crucial step in *Legionella* proliferation.

## Conflict of Interest

The authors declare no conflict of interest.

## Author Contributions

Assay optimalization, HTS, and validation were performed by **Halana C. Vlaming** with help from **Bjorn R. van Doodewaerd** and **Paul P. Geurink** under supervision of **Gerbrand J. van der Heden van Noort**. Synthesis of analogs **1**‐**4** was performed by Vito Pol. Protein expression was executed by **Angeliki Moutsiopoulou** under supervision of **Robbert Q. Kim**. The manuscript was prepared by **Halana C. Vlaming** and **Gerbrand J. van der Heden van Noort** with input from all authors.

## Supporting information

Supplementary Material

## Data Availability

The data that support the findings of this study are available in the supplementary material of this article.
