# Supplementary material for: Development and Validation of a High‐Throughput Screening Assay for the Legionella ADP‐Ribosyl Transferase SdeA
Source: Chembiochem. 2025 Sep 29;26(21):e202500513. doi: 10.1002/cbic.202500513 (PMC12596928; doi:10.1002/cbic.202500513)
Supplement: Supplementary file 1 — Supplementary Material [file CBIC-26-e202500513-s001.pdf]

Development and Validation of a High-Throughput Screening Assay for the Legionella ADP-Ribosyl Transferase SdeA

Halana C. Vlaming, Vito Pol, Bjorn R. van Doodewaerd, Angeliki Moutsiopoulou, Paul P. Geurink, Robbert Q. Kim, Gerbrand J. van der Heden van Noort*

Supporting Information

General synthetic procedures

Materials & Solvents

All reagents were used as received unless stated otherwise. Solvents used in synthesis were dried and stored over 4Å molecular sieves, except for MeOH and MeCN which were stored over 3Å molecular sieves. Column chromatography was performed on silica gel 60 Å (40-63 µm, Macherey-Nagel). TLC analysis was performed on Macherey-Nagel aluminium sheets (silica gel 60 F_{254}). TLC was used to visualize compounds by UV at wavelength 254 nm and by spraying with either cerium molybdate spray (25 g/L $(NH_4)_6Mo_7O_{24}$, 10 g/L $(NH_4)_4Ce(SO_4)_4 \cdot H_2O$ in 10% H_2SO_4 water solution) or $KMnO_4$ spray (20 g/L $KMnO_4$ and 10 g/L K_2CO_3 in water) followed by charring at c.a. 250 °C. NMR spectra were recorded on a Bruker AV-300 NMR. Chemical shifts (δ) are given in ppm relative to tetramethyl silane. Coupling constants (*J*) are given in Hz. All given ^{13}C-APT spectra are proton decoupled.

Analytical methods

LC-MS measurements and HPLC purifications

LC-MS measurements were conducted on a Waters ACQUITY UPLC H-class System equipped with a Waters ACQUITY Quaternary Solvent Manager (QSM), Waters ACQUITY UPLC Photodiode Array (PDA) eλ Detector (λ = 210-800 nm), Waters ACQUITY UPLC Protein BEH C18 column (1.7 µM, 2.1 x 50 mm) and LCT Premier Orthogonal acceleration Time of Flight Mass Spectrometer (m/z = 100-1600) in ES+ mode. Samples were run for 3 minutes at 40 °C using 2 mobile phases: A: MQ + 0.1% formic acid, B: MeCN + 0.1% formic acid. Gradient: 0 - 95% B at a flow rate of 0.5 mL/min.
High resolution mass spectra were recorded on a Waters XEVO-G2 XS Q-TOF mass spectrometer equipped with an electrospray ion source in positive mode (capillary voltage: 1.6 kV, desolvation gas flow: 900 L/h, temperature: 500 °C) with a resolution R = 22000 and 200 pg/µL Leu-Enk (m/z = 556.2771) as a "lock mass". Samples were run using 2 mobile phases: A = 0.1% formic acid in water and B = 0.1% formic acid in CH_3CN on a Waters Acquity UPLC BEH C18 column (2.1x50 mm, 1.7 µm); flow rate = 0.6 mL/min, runtime = 3.00 min, column T = 60 °C, mass detection: 50-1200 Da.

HPLC purifications were performed on **A**) a Waters HPLC equipped with a Waters 2489 UV/Vis detector and Waters fraction collector III using a reversed phase HPLC column as specified in the experimental section. Elution was performed using 2 mobile phases: A = 0.1 % TFA in MilliQ water and B = 0.1% TFA in acetonitrile using a linear gradient. Fractions were collected using a Gilson fraction collector and relevant fractions were assessed by analytical LC-MS. Fractions containing the pure peptide were pooled and lyophilized. **B**) a Waters LC Prep Autopurification System. Compound purification was performed on a Waters C18-Xbridge 5 µm OBD (19 x 150 mm) column. Compounds were loaded onto the column at 1 mL/min using an at column dilution setup CH_3CN with 1.5% TFA was used as carrier liquid. Using a gradient of A: MilliQ water and B: CH_3CN on a gradient of 0.5% to 20% B over 17.5 min with a flowrate of 30 mL/min separation was achieved.

Size Exclusion Chromatography

After HPLC purification lyophilized fractions containing Ub were refolded by gel filtration using a size exclusion S75 16/600 superdex PG-GE healthcare column with a volumebed of 120 mL and 3-70 kDa separation range using a filtered aqueous buffer containing 50 mM TRIS 50 mM NaCl, pH 7.5 at a flowrate of 1 mL/min on a Biorad NGC Chromatography system. The sample was prepared by dissolving the lyophilized Ub in DMSO (150 µL) followed by dropwise addition of this solution into MilliQ (1.5 mL). This solution was added dropwise to filtered buffer containing 50 mM TRIS, 50 mM NaCl, pH 7.5. The mixture was centrifuged for 5 minutes at 1500 rpm. The appropriate NGC fractions were analysed by SDS-PAGE and pure fractions were pooled, combined and concentrated using a 3kDa cut-off spin filter.

Protein Expression and Purification

SdeA construct (resideus 231-1190) containing both PDE and mART domains was produced according to Kim *et al*.[1] In short, pET22b_SdeA-PDE-mART-CC was transformed into BL21(DE3) cells and plated on LB agar containing Ampicillin as a selection marker. A single colony was used to inoculate an overnight preculture before large-scale growth in LB. Cultures were grown until an OD_{600} of 0.9 and subsequently overnight induced at 18°C using 0.4 mM Isopropyl β-D-1-thiogalactopyranoside. Spun-down (4kG, 20 min) cells were resuspended in His buffer A (20 mM Tris pH 8.0, 300 mM NaCl and 20 mM Imidazole) and lysed using sonication on ice. Every subsequent step was carried out at 4°C, starting with centrifugation at 24 kG for 40 min. The resulting supernatant was applied to NiNTA beads, preequilibrated in His buffer A. After being thoroughly (100 CV) washed with buffer A, protein was eluted with buffer A supplemented with 200 mM Imidazole. Fraction were assessed on SDS-PAGE and SdeA-protein-containing ones were pooled and three-fold diluted using buffer Q-A (25 mM Tris pH8.0). This sample was loaded on a 1 mL HiTrap Q (Cytiva) column, equilibrated using buffer Q-A) and using a gradient with buffer Q-B (25 mM Tris pH8.0, 1 M NaCl) SdeA protein could be separated, as assessed by SDS-PAGE. As a final polishing step, SdeA fractions were pooled and run on a Superdex200 16/60 column in 20 mM Tris pH8.0, 150 mM NaCl) using an NGC chromatography system (Biorad). The peak containing SdeA was collected, concentrated and flash-frozen using liquid N_2 for storage at -70°C.

Chemical synthesis

Solid Phase Peptide Synthesis (SPPS)

SPPS was performed on a SYRO II (Multisyntech, SYRO Robot) automated peptide synthesizer using standard 9-fluorenylmethoxycarbonyl (Fmoc) based SPPS. Fmoc deprotection was achieved with 2 x 2 min. and 1x 5 min treatment of 20 vol. % piperidine in NMP. Peptide couplings were performed using PyBOP/DIPEA. Amino acid solutions (0.34 M in NMP) were added to the resin at 4-fold excess together with an 8-fold excess of DIPEA (1.36 M in NMP) and 4-fold excess of PyBOP (0.34 M in NMP). The coupling time was 2x 25 minutes unless specified otherwise. After the final Fmoc deprotection the resin was washed with NMP and DCM and Et_2O.

Synthesis of Ub$_{1-76}$

The synthesis reported by Oualid *et al*. was closely followed.[2] The product was repurified by gel filtration using a Biorad NGC Chromatography system on a size exclusion following general procedures.

Table S1.

Segment ID	SPPS sequence
Ub$_{1-76}$ (Ub)	XQIFVKTLTGKTITLEVEPSDTIENVKAKIQDKEGIPPDQQRLIFAGKQLEDGRTLSDYNIQ KESTLHLVLRLRGG

Amino acid sequence of synthesized Ub, pseudoprolines are underscored. X was incorporated as a Fmoc-norleucine-OH. Underlined dipeptide sequences were coupled as the respective pseudoproline dipeptides or DMB dipeptides.

Purification of expressed Ub$_{1-76}$

Ubiquitin wild-type was purified by lysing the cell pellet in MilliQ through heat denaturation (85 °C, 30 minutes). After cooling the suspension, DNA was digested by adding 15 µg/mL DNase I (Roche) and 10 mM MgCl2 for 15 minutes at 4 °C. The suspension was heated again to 85 °C for 30 minutes to denature DNase I, cooled down and centrifuged at 15,000 g for 30 minutes. The supernatant was purified by cation exchange chromatography on a custom-made column (WorkBeads 40S; BioWorks) and purified over a gradient of 0 – 0.6 M NaCl in 50 mM NaOAc pH 4.6 over 15 column volumes. Appropriate fractions were pooled, concentrated and subjected to a S75 16/60 column (GE healthcare) containing 20 mM Tris-HCl pH 7.6 and 150 mM NaCl. Fractions were analyzed by SDS-PAGE and Coomassie Brilliant Blue staining after which pure protein was concentrated and stored at -20 °C for further use.

Synthesis of ε-NAD

The synthesis reported by Barrio *et al* was closely followed.[3]

N-succinimidyl octanoate

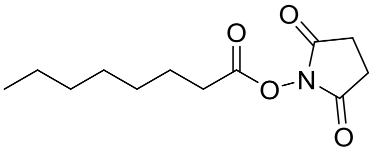

Octanoic acid (0.3 mL, 2 mmol, 1 eq) was dissolved in anhydrous DCM (8 mL), followed by addition of N-Hydroxysuccinimidyl (345 mg, 3 mmol, 1.5 eq) resulting in a suspension. The mixture was cooled to 0 °C followed by addition of 1-ethyl-3-(3-dimethylaminopropyl)carbodiimide (EDC) (575 mg, 3 mmol, 1.5 eq) and 4-dimethylaminopyridine (DMAP) (36.6 mg, 0.3 mmol, 0.1 eq). The resulting solution was allowed to go to r.t after 5 min stirring at 0 °C, and was stirred overnight. Complete conversion was shown using TLC-analysis. The solution was extracted using 1M HCl (2x) and bicarb (1x). The organic layers were combined and dried over $MgSO_4$, filtered using vacuum filtration and concentrated under reduced pressure. The

crude was purified by silica gel column chromatography (heptane/EtOAc= 75:25) affording **N-succinimidyl octanoate** (416 mg, 1.72 mmol, 86% yield) as a white solid. ^{1}H NMR (300 MHz, Chloroform-*d*) δ 2.82 (d, 4H), 2.59 (t, *J* = 7.5 Hz, 2H), 1.87 – 1.59 (m, 2H), 1.45 – 1.12 (m, 8H), 0.96 – 0.71 (m, 3H). ^{13}C NMR (75 MHz, Chloroform-*d*) δ 169.32, 168.82, 31.65, 31.07, 28.86, 25.72, 24.70, 22.67, 14.16.

Synthesis of cephalosporin analogues 1-4

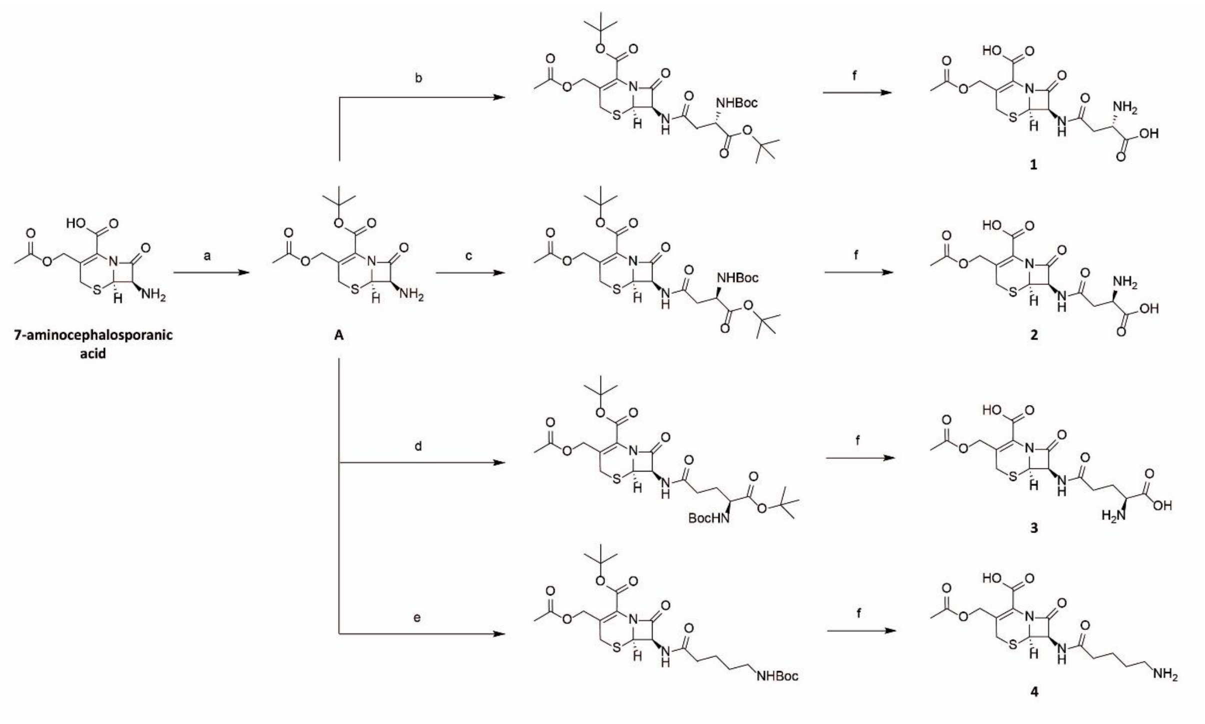

Scheme S1. Synthesis route of cephalosporin analogues (**1**-**4**). Reagents and conditions: (a) *tert*-butyl acetate, BF_3Et_2O/ literature procedure[4] (b) Boc-L-aspartic acid a-*tert*-butyl ester, EDC, HOBt, DIPEA, DMF, r.t. overnight; (c) Boc-D-aspartic acid a-*tert*-butyl ester, EDC, HOBt, DIPEA, DMF, r.t. overnight; (d) Boc-L-glutamic acid a-tert butyl ester, EDC, HOBt, DIPEA, DMF, r.t. overnight; (e) 5-(Boc-amino)valeric acid, , EDC, HOBt, DIPEA, DMF, r.t. overnight; (f) HCl/dioxane, r.t.

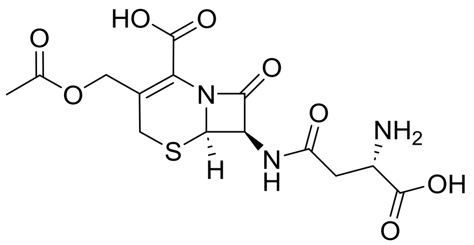

7-aminocephalosporanic acid – (L) aspartic acid analogue (1)

7-aminocephalosporanic acid *t*-butyl ester[4] (40.0 mg, 0.12 mmol, 1 eq,) was dissolved in DMF (1 mL) followed by addition of Boc-L-aspartic acid a-*tert*-butyl ester, 1-ethyl-3-(3-dimethylaminopropyl)carbodiimide (EDC) (35.0 mg, 0.15 mmol, 1.5 eq), HOBt (24.7 mg, 1.5 eq, 0.18 mmol) and DIPEA (47.2 mg, 3 eq, 0.06 mL) and was stirred over night. After LC-MS analysis revealed full conversion the crude was concentrated under reduced pressure, and was treated with HCl / DCM (1:1) until full deprotection was reached shown by LC-MS analysis. The crude was concentrated under reduced pressure and dissolved in DMSO, followed using preparatory HPLC, affording compound **1** (10.38 mg, 0.027 mmol, 22% yield. ^{1}H NMR (300 MHz, Methanol-d_4) δ 5.79 (d, *J* = 4.8 Hz, 1H), 5.14 – 5.02 (m, 2H), 4.88 – 4.82 (m, 1H), 4.82 (s, 1H), 4.11 – 4.01 (m, 1H), 3.69 – 3.41 (m, 2H), 3.06 (dd, *J* = 17.2, 3.8 Hz, 1H), 2.88 (dd, *J* = 17.2, 7.9 Hz, 1H), 2.06 (s, 3H).^{13}C NMR (75 MHz, Methanol-d_4) δ 172.29, 166.07, 124.57, 64.60, 60.32, 58.68, 49.00 (dp, *J* =

42.8, 21.4 Hz), 35.53, 27.06, 20.62. HRMS calculated for $[C_{14}H_{17}N_3O_8S + H]^+$: 388.0815, found: 388.0808.

7-aminocephalosporanic acid – (D) aspartic acid analogue (2)

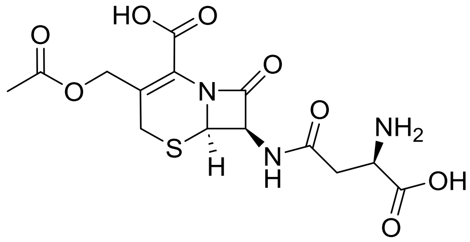

7-aminocephalosporanic acid *t*-butyl ester (40.0 mg, 0.12 mmol, 1 eq,) was dissolved in DMF (1 mL) followed by addition of Boc-D-aspartic acid a-*tert*-butyl ester, 1-ethyl-3-(3-dimethylaminopropyl)carbodiimide (EDC) (35.0 mg, 0.15 mmol, 1.5 eq), HOBt (24.7 mg, 1.5 eq, 0.18 mmol) and DIPEA (47.2 mg, 3 eq, 0.06 mL) and was stirred over night. After LC-MS analysis revealed full conversion the crude was concentrated under reduced pressure, and was treated with HCl / DCM (1:1) until full deprotection was reached shown LC-MS analysis. The crude was concentrated under reduced pressure and dissolved in DMSO and purified by preparatory HPLC affording aspartate (D) analogue **2** (9.95 mg, 0.026 mmol, 21% yield). ^{1}H NMR (300 MHz, Methanol-d_4) δ 5.79 (d, J = 4.9 Hz, 1H), 5.13 – 5.06 (m, 2H), 4.88 (s, 46H), 4.03 (dd, J = 9.2, 3.4 Hz, 1H), 3.70 – 3.41 (m, 2H), 3.05 (dd, J = 17.3, 3.5 Hz, 1H), 2.83 (dd, J = 17.3, 9.2 Hz, 1H), 2.06 (s, 3H). ^{13}C NMR (75 MHz, Methanol-d_4) δ 172.35, 166.01, 124.60, 64.60, 60.40, 58.73, 35.87, 27.06, 20.60. HRMS calculated for $[C_{14}H_{17}N_3O_8S + H]^+$: 388.0815, found: 388.0798.

7-aminocephalosporanic acid – (L) glutamine analogue (3)

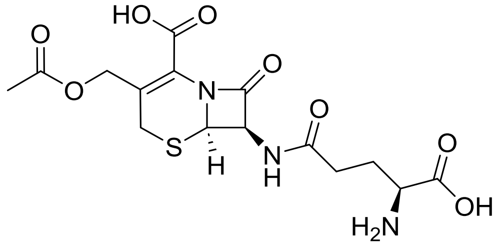

7-aminocephalosporanic acid *t*-butyl ester (40.0 mg, 0.12 mmol, 1 eq,) was dissolved in DMF (1 mL) followed by addition of Boc-L-glutamic acid a-tert butyl ester, 1-ethyl-3-(3-dimethylaminopropyl)carbodiimide (EDC) (35.0 mg, 0.15 mmol, 1.5 eq), HOBt (24.7 mg, 1.5 eq, 0.18 mmol) and DIPEA (47.2 mg, 3 eq, 0.06 mL) and was stirred over night. After LC-MS analysis revealed full conversion the crude was concentrated under reduced pressure, and was treated with HCl / DCM (1:1) until full deprotection was reached shown by LC-MS analysis. The crude was concentrated under reduced pressure and dissolved in DMSO and purified by preparatory HPLC, affording glutamate analogue **3** (8.01 mg, 0.020 mmol, 17% yield). ^{1}H NMR (300 MHz, DMSO-d_6) δ 9.00 (d, J = 8.2 Hz, 1H), 5.69 (dd, J = 8.2, 4.8 Hz, 1H), 5.10 (d, J = 4.9 Hz, 1H), 4.84 (dd, J = 94.3, 12.8 Hz, 2H), 3.92 (t, J = 6.4 Hz, 1H), 3.56 (dd, 2H), 2.44 – 2.33 (m, 1H), 2.03 (m, 5H). ^{13}C NMR (75 MHz, DMSO-d_6) δ 171.71, 170.74, 170.21, 164.68, 162.85, 126.41, 123.40, 62.72, 59.03, 57.32, 51.59, 30.17, 25.88, 25.57, 20.60. HRMS calculated for $[C_{15}H_{19}N_3O_8S + H]^+$: 402.0971, found: 402.0965.

7-aminocephalosporanic acid – aminovaleric acid (4)

7-aminocephalosporanic acid *t*-butyl ester (40.0 mg, 0.12 mmol, 1 eq,) was dissolved in DMF (1 mL) followed by addition of 5-(Boc-amino)valeric acid, 1-ethyl-3-(3-dimethylaminopropyl)carbodiimide (EDC) (35.0 mg, 0.15 mmol, 1.5 eq), HOBt (24.7 mg, 1.5 eq, 0.18 mmol) and DIPEA (47.2 mg, 3 eq,

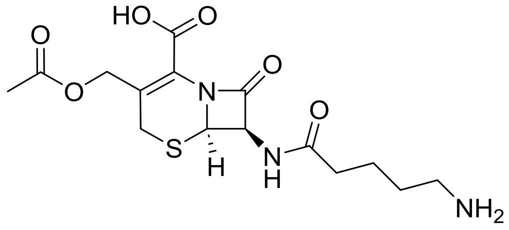

0.06 mL) and was stirred over night. After LC-MS analysis revealed full conversion the crude was concentrated under reduced pressure, and was treated with HCl / DCM (1:1) until full deprotection was reached shown by LC-MS analysis. The crude was concentrated under reduced pressure and dissolved in DMSO and purified by preparatory HPLC, affording aminovaleric analogue **4** (4.94 mg, 0.013 mmol, 11% yield). ^{1}H NMR (300 MHz, DMSO-*d6*) δ 8.83 (d, *J* = 8.2 Hz, 1H), 5.67 (dd, *J* = 8.2, 4.8 Hz, 1H), 5.08 (d, *J* = 4.8 Hz, 1H), 4.84 (dd, *J* = 92.8, 12.7 Hz, 2H), 3.58 (dd, 2H), 2.84 – 2.71 (m, 2H), 2.31 – 2.17 (m, 2H), 1.64 – 1.48 (m, 4H). HRMS calculated for $[C_{15}H_{21}N_3O_6S + H^+]$: 372.1229, found: 372.1224.

Assays

Formula used to calculate Z':

$$Z' = 1 - \frac{3 \cdot (\delta_{neg\ control} + \delta_{pos\ control})}{|\mu_{neg\ control} - \mu_{pos\ control}|}$$

δ: standard deviation

µ: average

SdeA activity measurements with substrate ε-NAD/Ub

The assay was performed in a flat-bottom black 384-well plate (Corning 3820) at room temperature in a buffer containing 50 mM TRIS, 50 mM NaCl, pH 7.5. ε-NAD/Ub (8 µL, 50 µM final concentration in a 1:1 ratio) was added to a serial dilution of SdeA (4 µL, 50 nM, 20 nM, 10 nM, 5 nM final concentration) using a multichannel pipette. Either synthetic $Ub_{1\text{-}76}$ or expressed $Ub_{1\text{-}76}$ were used. The plate was centrifuged at (500 rpm, 1 minute) followed by recording the Fluorescence Intensity (FI) for 1 hour in intervals of 2 minutes on a BMG Labtech PHERAstar plate reader ($\lambda_{ex}/_{em}$ = 320/380).

Testing N-succinimidyl octanoate as positive control.

The assay was performed in a flat-bottom black 384-well plate (Corning 3820) at room temperature in a buffer containing 50 mM TRIS, 50 mM NaCl, pH 7.5. All was dispensed using a multichannel pipet. A serial dilution of N-succinimidyl octanoate stocks (5 mM, 2.5 mM, 1.25 mM, 0.625 mM, 0.313 mM, 0.157 mM, 0.078 mM, 0.039 mM final concentrations) was prepared in DMSO and was added to SdeA (4 µL, 10 nM) followed by incubation for 30 minutes at room temperature. Next, Ub (4 µL, 50 µM final concentration) and ε-NAD (4 µL, 50 µM final concentration) was added, followed by recording the fluorescence Intensity (FI) on a BMG Labtech CLARIOstar plate reader ($\lambda_{ex}/_{em}$ = 330/410).

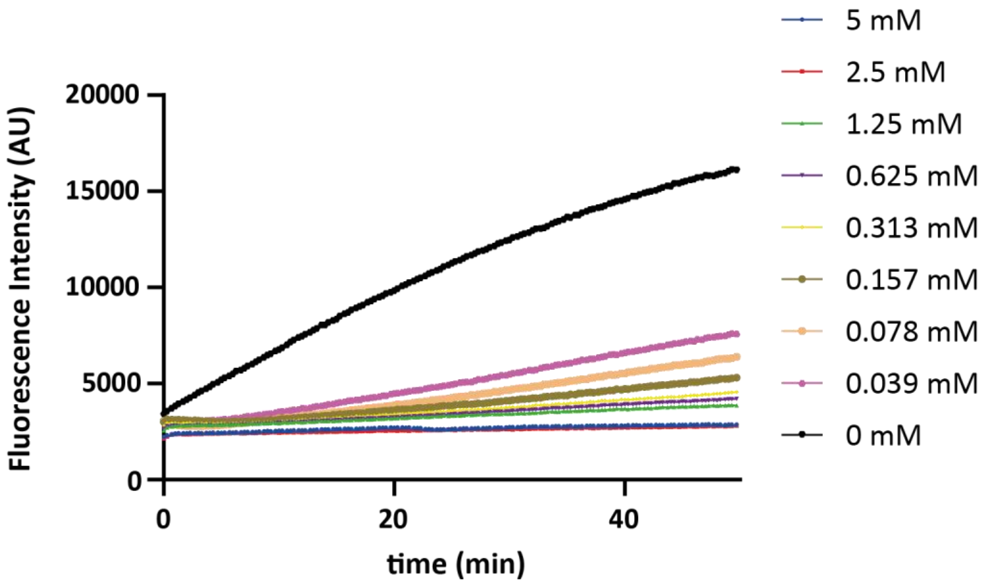

Figure S1. SdeA activity at different N-succinimidyl octanoate concentrations.

Replicates measurement of control

The assay was performed in a flat-bottom, low-flange, black 384-well plate (Corning 3820) at room temperature in a buffer 50 mM TRIS, 50 mM NaCl, pH 7.5. Octanoic acid *N*-hydroxysuccinimidyl ester (1.25 mM final concentration) was used as a positive control (100% inhibition) and DMSO was used as a negative control (0% inhibition). Both were transferred from DMSO stocks (200 nL) to an empty 'destination' plate using a Labcyte Echo550 acoustic dispenser, filling 96 wells for the positive control and 96 wells for the negative control. Buffered solutions were dispensed using a Biotek MultiFlowFX liquid dispenser. Next, buffer (10 µL) was dispensed and the plate was shaken gently for 10 seconds, centrifuged (500 rpm, 1 min), followed by dispensing SdeA (5 µL, final concentration of 10 nM) and incubated for 30 minutes at room temperature. Afterwards substrate ε-NAD/Ub (1:1) was dispensed (5 µL, final concentration of 50 µM) and the plate was centrifuged again (500 rpm, 1 min), followed by recording the Fluorescence Intensity (FI) on a BMG Labtech CLARIOstar plate reader ($\lambda_{ex}/_{em}$ = 330/410).

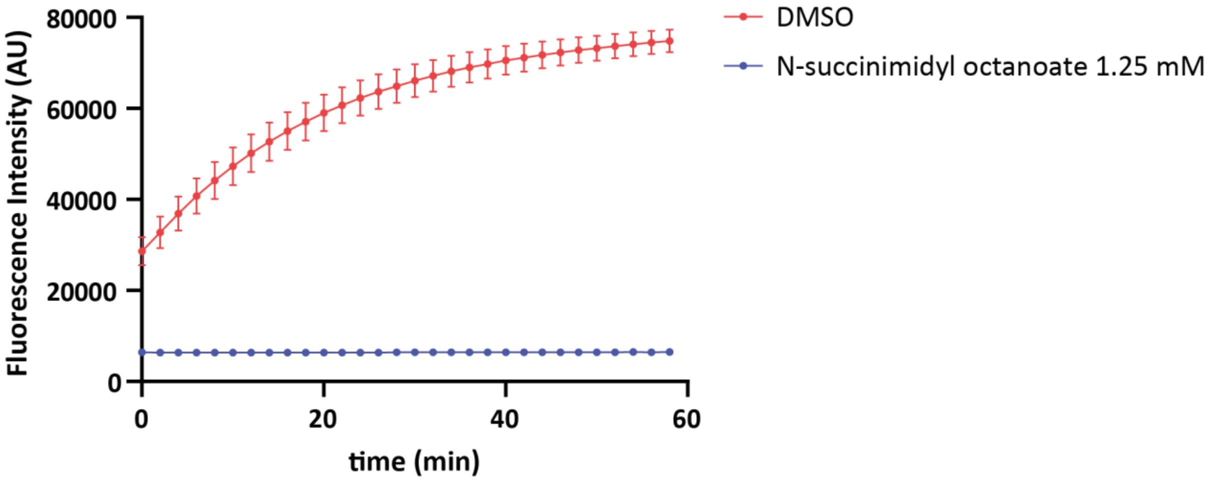

Figure S2. ADP-ribosylation of Ub with ε-NAD catalyzed by enzyme SdeA (10 nM) treated with DMSO (red line, negative control) showing SdeA activity, and treated with N-succinimidyl octanoate (blue line) causing inhibition at 1.25 mM.

High-Throughput Screening

High-Throughput screening was performed in flat-bottom, low-flange, black 384-well plates (Corning 3820) at room temperature in a buffer 50 mM TRIS 50 mM NaCl, pH 7.5. Octanoic acid *N*-hydroxysuccinimidyl ester (1.25 mM final concentration) was used as a positive control (100% inhibition) and DMSO was used as a negative control (0% inhibition). Both were transferred from DMSO stocks (200 nL) to an empty 'destination' plate using a Labcyte Echo550 acoustic dispenser. Buffered solutions were dispensed using a Biotek MultiFlowFX liquid dispenser. The compound library 'Library Of Pharmacologically Active Compounds (LOPAC)' containing 640 compounds dissolved in DMSO were transferred from a source plate into an empty 384 well plate (200 nL) to obtain a 5 µM final compound concentration. Next, buffer (10 µL) was dispensed and the plate was shaken gently for 10 seconds, centrifuged (500 rpm, 1 min), followed by dispensing SdeA (5 µL, final concentration of 10 nM) and incubated for 30 minutes at room temperature. Afterwards substrate ε-NAD/Ub (1:1) was dispensed (5 µL, final concentration of 50 µM) and the plate was centrifuged again (500 rpm, 1 min), and the plate was incubated for 28 minutes, followed by recording the Fluorescence Intensity (FI) on a BMG Labtech CLARIOstar plate reader ($\lambda_{ex}/_{em}$ = 330/410). The percentage of inhibition was calculated by normalizing these to the positive and negative controles, using the following formula:

$$\%\,inh = 100 * (1 - \frac{(X - \mu_{pos\ control})}{(\mu_{neg\ control} - \mu_{pos\ control})})$$

µ: average of FI values

X: FI value of measured sample

IC_{50} determination of cephalosporin C Zn^{2+} salt

The assay was performed in a flat-bottom, low-flange, black 384-well plate (Corning 3820) at room temperature in buffer 50 mM TRIS 50 mM NaCl, pH 7.5 with a reaction volume of 20 µL in triplicate. Stock solutions of cephalosporin C Zn^{2+} salt of 0.1 mM, 1 mM and 10 mM were prepared in DMSO. The compounds were transferred to an empty 'destination' plate using a Labcyte Echo550 acoustic dispenser to obtain a 8-point serial dilution of 0.037 to 100 µM. Octanoic acid *N*-hydroxysuccinimidyl ester (1.25 mM final concentration) was used as a positive control (100% inhibition) and DMSO was used as a negative control (0% inhibition). Buffered solutions were dispensed using a Biotek MultiFlowFX liquid dispenser. Next, buffer (10 µL) was dispensed and the plate was shaken gently for 10 seconds, centrifuged (500 rpm, 1 min), followed by dispensing SdeA (5 µL, final concentration of 10 nM) and incubated for 30 minutes at room temperature. Afterwards substrate ε-NAD/Ub (1:1) was dispensed (5 µL, final concentration of 50 µM) followed by recording the Fluorescence Intensity (FI) on a BMG Labtech PHERAstar plate reader ($\lambda_{ex}/_{em}$ = 320/380). Then the slope of the linear part of the reaction curve was calculated, and from this the percentage of inhibition was calculated by normalizing these to the positive and negative controles. These values were plotted against the logarithmic values of the inhibitor concentrations (in nM) and from this the IC_{50} values were obtained using the built-in equation "[inhibitor] vs response variable slope (four parameters), least-squares fit" with constraints "Bottom = 0"and "Top = 100"in GraphPad Prism 8.4.2 software.

$$\% \, inh = 100 - 100 * (\frac{(X - \mu_{pos\ control})}{(\mu_{neg\ control} - \mu_{pos\ control})})$$

µ: average of slope

X: FI value of slope

IC_{50} determination of cephalosporin analogues 1-4 without $ZnCl_2$ and with $ZnCl_2$.

The assay was performed in a flat-bottom, low-flange, black 384-well plate (Corning 3820) at room temperature in buffer 50 mM TRIS, 50 mM NaCl, pH 7.5 with a reaction volume of 20 µL in triplicate. Stock solutions of the compounds of 0.1 mM and 10 mM were prepared in DMSO. Cephalosporin analogues with $ZnCl_2$ were mixed in a 1:1 ratio by first preparing a 100mM solution of $ZnCl_2$ in MQ, which was further diluted in DMSO obtaining the correct dilution. The compounds were transferred to an empty 'destination' plate using a Labcyte Echo550 acoustic dispenser to obtain a 8-point serial dilution of 0.037 to 100 µM. Octanoic acid *N*-hydroxysuccinimidyl ester (1.25 mM final concentration) was used as a positive control (100% inhibition) and DMSO was used as a negative control (0% inhibition). Buffered solutions were dispensed using a Biotek MultiFlowFX liquid dispenser. Next, buffer (10 µL) was dispensed and the plate was shaken gently for 10 seconds, centrifuged (500 rpm, 1 min), followed by dispensing SdeA (5 µL, final concentration of 10 nM) and incubated for 30 minutes at room temperature. Afterwards substrate ε-NAD/Ub (1:1) was dispensed (5 µL, final concentration of 50 µM) followed by measuring the Fluorescence Intensity (FI) on a BMG Labtech PHERAstar plate reader ($\lambda_{ex}/_{em}$ = 320/380) for 1 hour. Then the slope of the linear part of the reaction curve was calculated, and from this the percentage of inhibition was calculated by

normalizing these to the positive and negative controles. These values were plotted against the logarithmic values of the inhibitor concentrations (in nM) and from this the IC_{50} values were obtained using the built-in equation “[inhibitor] vs response variable slope (four parameters), least-squares fit” with constraints “Bottom = 0”and “Top = 100”in GraphPad Prism 8.4.2 software.

$$\% \, inh = 100 - 100 * (\frac{(X - \mu_{pos\ control})}{(\mu_{neg\ control} - \mu_{pos\ control})})$$

μ: average of slope

X: FI value of slope

IC_{50} determination of bivalent metal salts $CuCl_2$, $MgCl_2$, $MnCl_2$, $NiCl_2$, $CoCl_2$, $Pb(NO_3)_2$, $Cd(NO_3)_2$, $ZnCl_2$.

The assay was performed in a flat-bottom, low-flange, black 384-well plate (Corning 3820) at room temperature in buffer 50 mM TRIS, 50 mM NaCl, pH 7.5 with a reaction volume of 20 µL in triplicate. The bivalent metals were first dissolved in MQ resulting in a 100mM solution, which were further dissolved in DMSO obtaining the correct dilution. Stock solutions of the compounds of 0.001 mM, 0.1 mM and 10 mM were prepared in DMSO. The compounds were transferred to an empty ‘destination’ plate using a Labcyte Echo550 acoustic dispenser to obtain a 8-point serial dilution of 0.037 to 100 µM. Octanoic acid *N*-hydroxysuccinimidyl ester (1.25 mM final concentration) was used as a positive control (100% inhibition) and DMSO was used as a negative control (0% inhibition). Buffered solutions were dispensed using a Biotek MultiFlowFX liquid dispenser. Next, buffer (10 µL) was dispensed and the plate was shaken gently for 10 seconds, centrifuged (500 rpm, 1 min), followed by dispensing SdeA (5 µL, final concentration of 10 nM) and incubated for 30 minutes at room temperature. Afterwards substrate ε-NAD/Ub (1:1) was dispensed (5 µL, final concentration of 50 µM) followed by measuring the Fluorescence Intensity (FI) on a BMG Labtech PHERAstar plate reader (λ_{ex} /$_{em}$ = 320/380) for 1 hour. Then the slope of the linear part of the reaction curve was calculated, and from this the percentage of inhibition was calculated by normalizing these to the positive and negative controles. These values were plotted against the logarithmic values of the inhibitor concentrations (in nM) and from this the IC_{50} values were obtained using the built-in equation “[inhibitor] vs response variable slope (four parameters), least-squares fit” with constraints “Bottom = 0”and “Top = 100”in GraphPad Prism 8.4.2 software.

$$\% \, inh = 100 - 100 * (\frac{(X - \mu_{pos\ control})}{(\mu_{neg\ control} - \mu_{pos\ control})})$$

μ: average of slope

X: FI value of slope

EDTA assay

The assay was performed in a flat-bottom black 384-well plate (Corning 3820) at room temperature in a buffer containing 50 mM TRIS 50 mM NaCl, pH 7.5 with a reaction volume of 20 µL in triplicate. All was dispensed using a multichannel pipet. A serial dilution of EDTA stocks (20 µM, 10 µM, 5 µM, 2.5 µM, 1.25 µM, 0.625 µM final concentrations) was prepared in buffer (19 µL), followed by addition of cephalosporin C Zn^{2+} salt in DMSO (1 µL, 5 µM final concentration) and was incubated for 30 minutes at room temperature. Next, 5 µL of these stock solutions was transferred into the wells and SdeA (10 µL) was added, centrifuged (500 rpm, 30 seconds) followed by 30 minutes incubation at room temperature. Next, ε-NAD/Ub (5 µL, 50 µM final concentration in a 1:1 ratio) was added, followed by centrifuging at 500 rpm for 30 seconds and the plate was the Fluorescence Intensity (FI) was measured on a BMG Labtech PHERAstar plate reader ($\lambda_{ex}/_{em}$ = 320/380).

NMR data:

N-succinimidyl octanoate

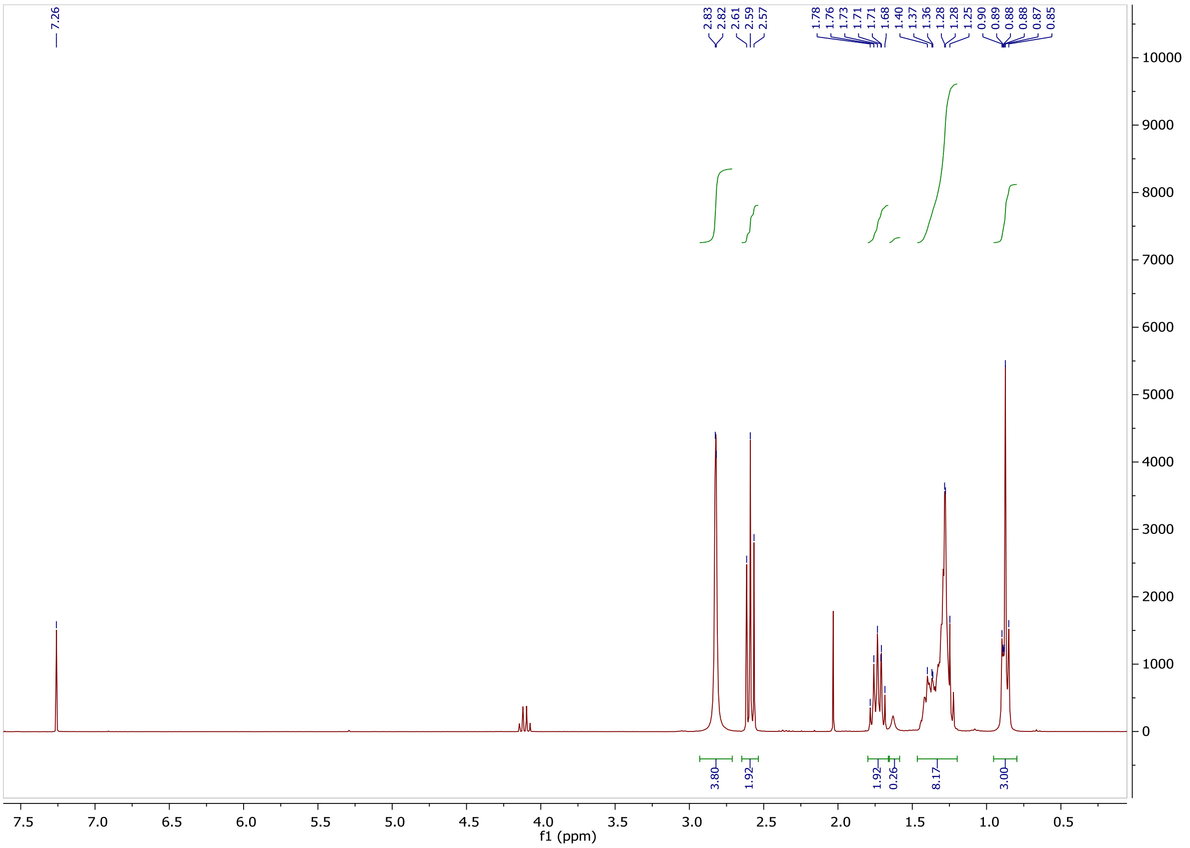

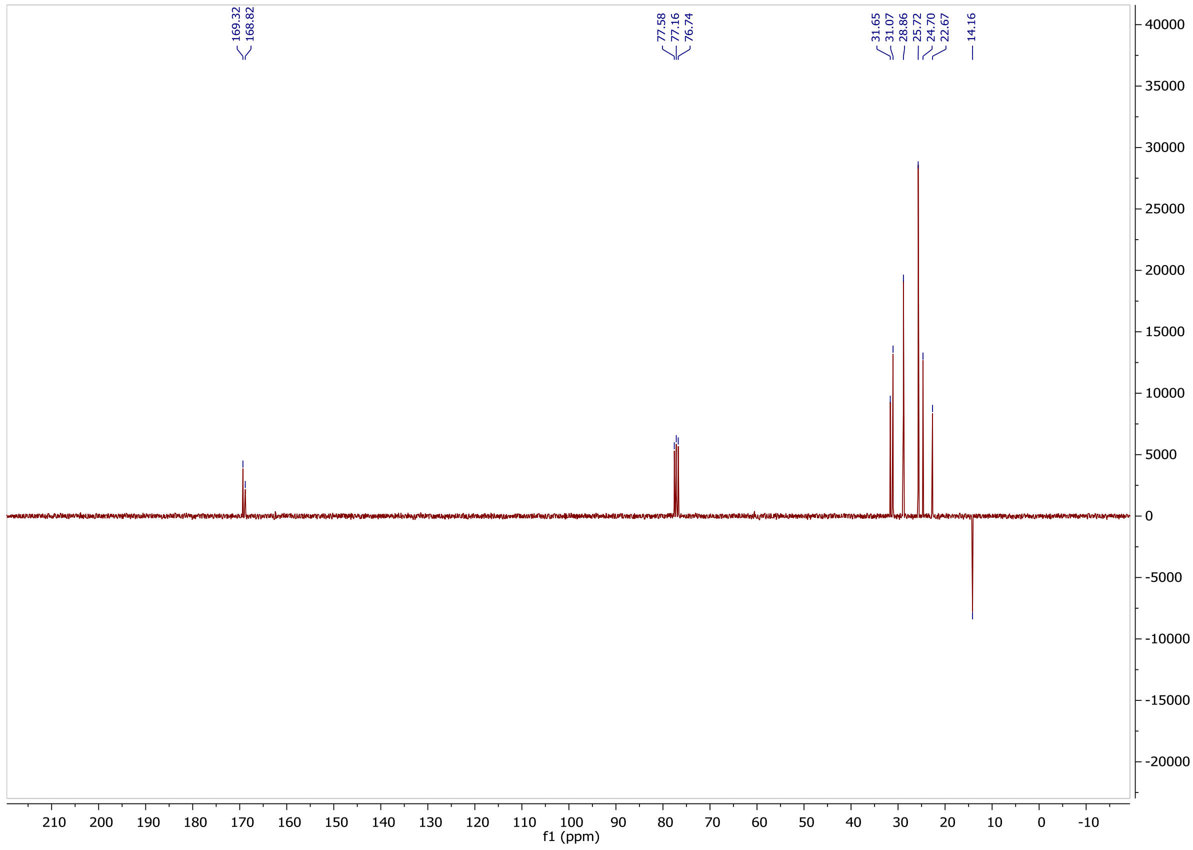

7-aminocephalosporanic acid – aspartate (L) analogue (1)

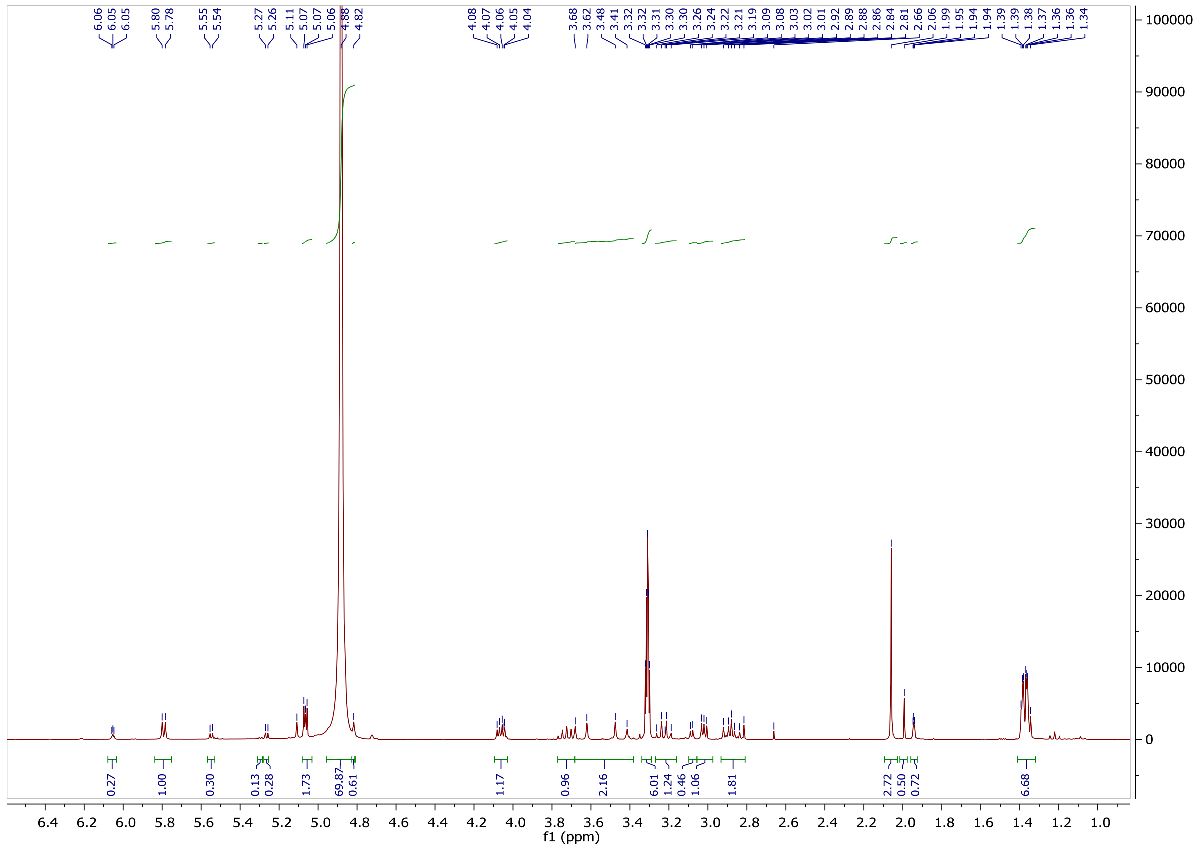

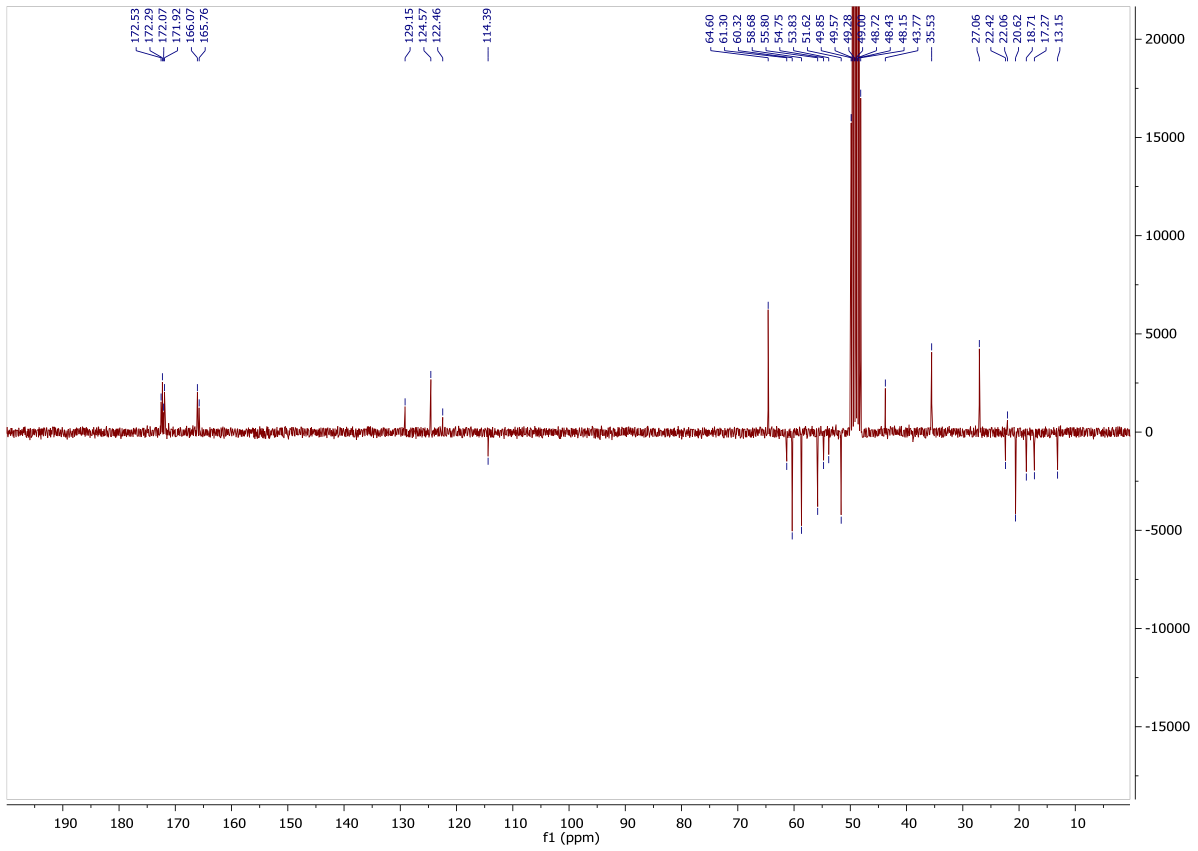

7-aminocephalosporanic acid – aspartate (D) analogue (2)

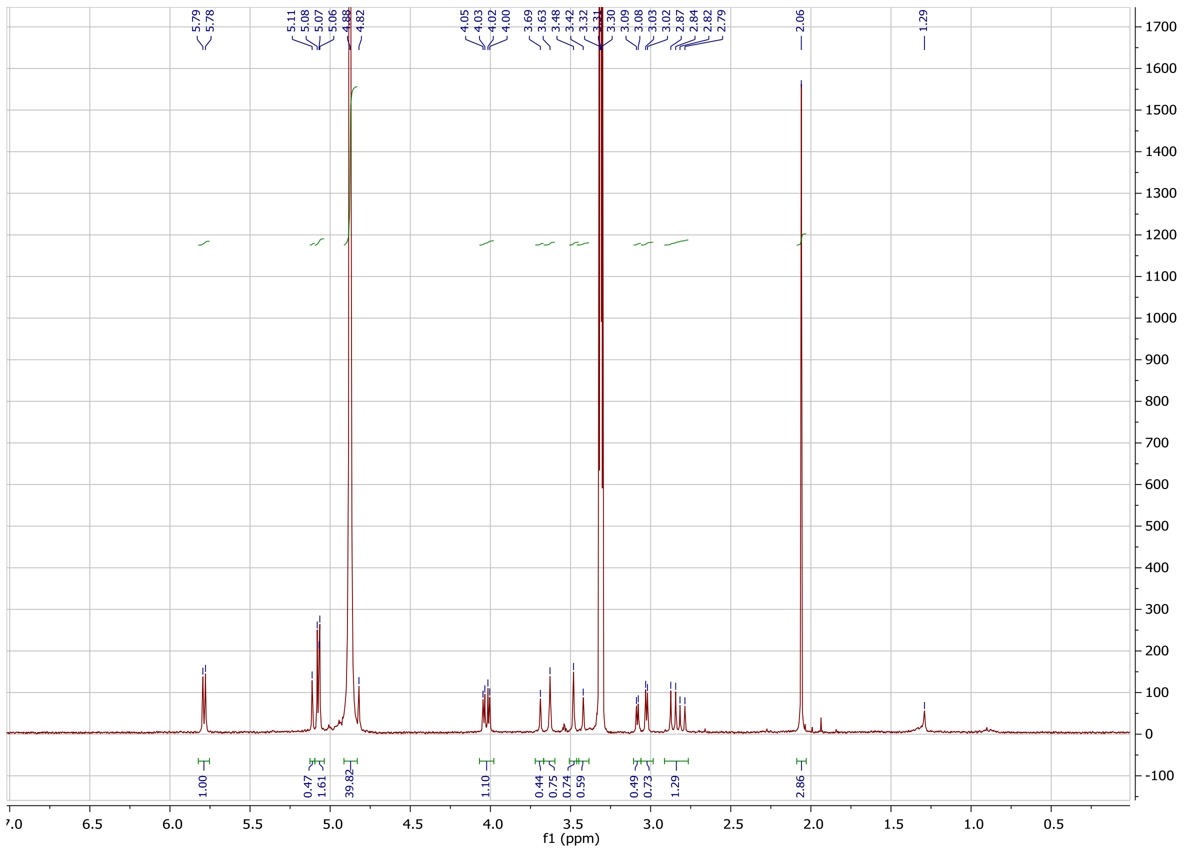

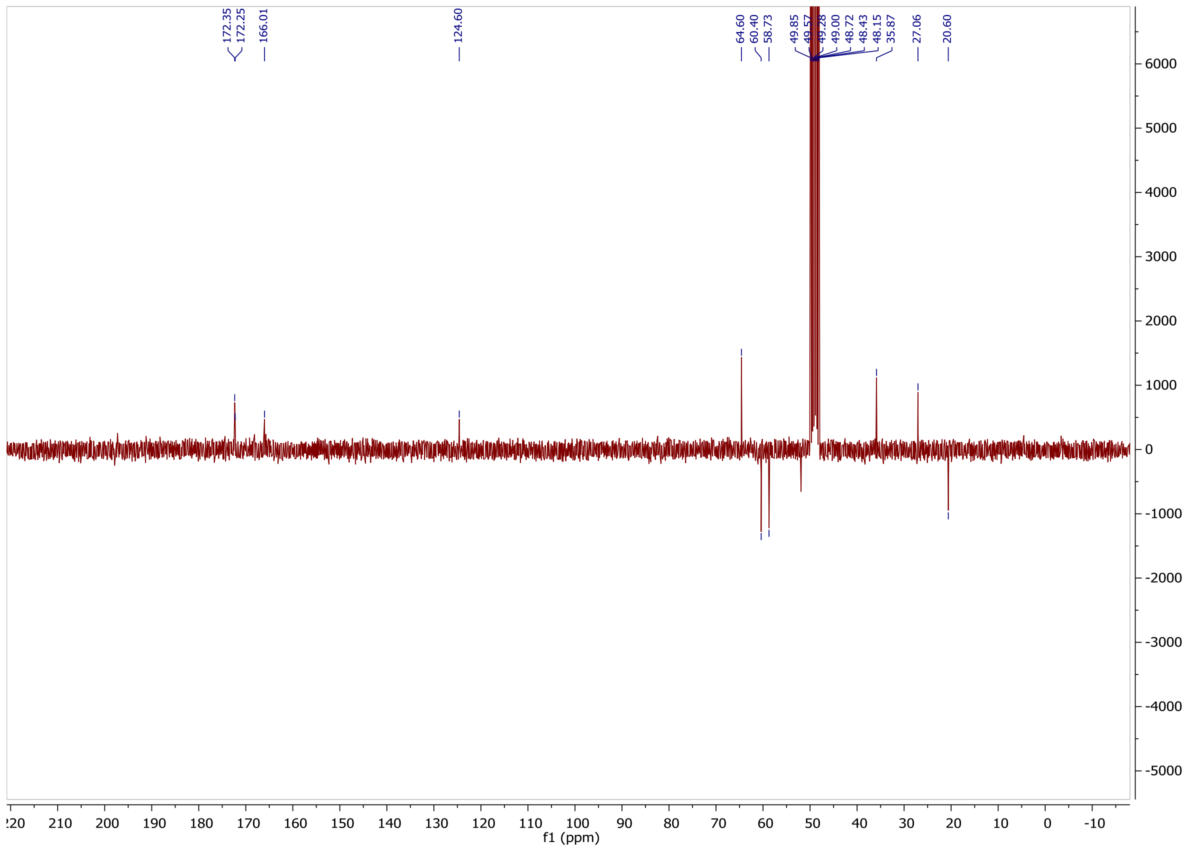

7-aminocephalosporanic acid – glutamine analogue (3)

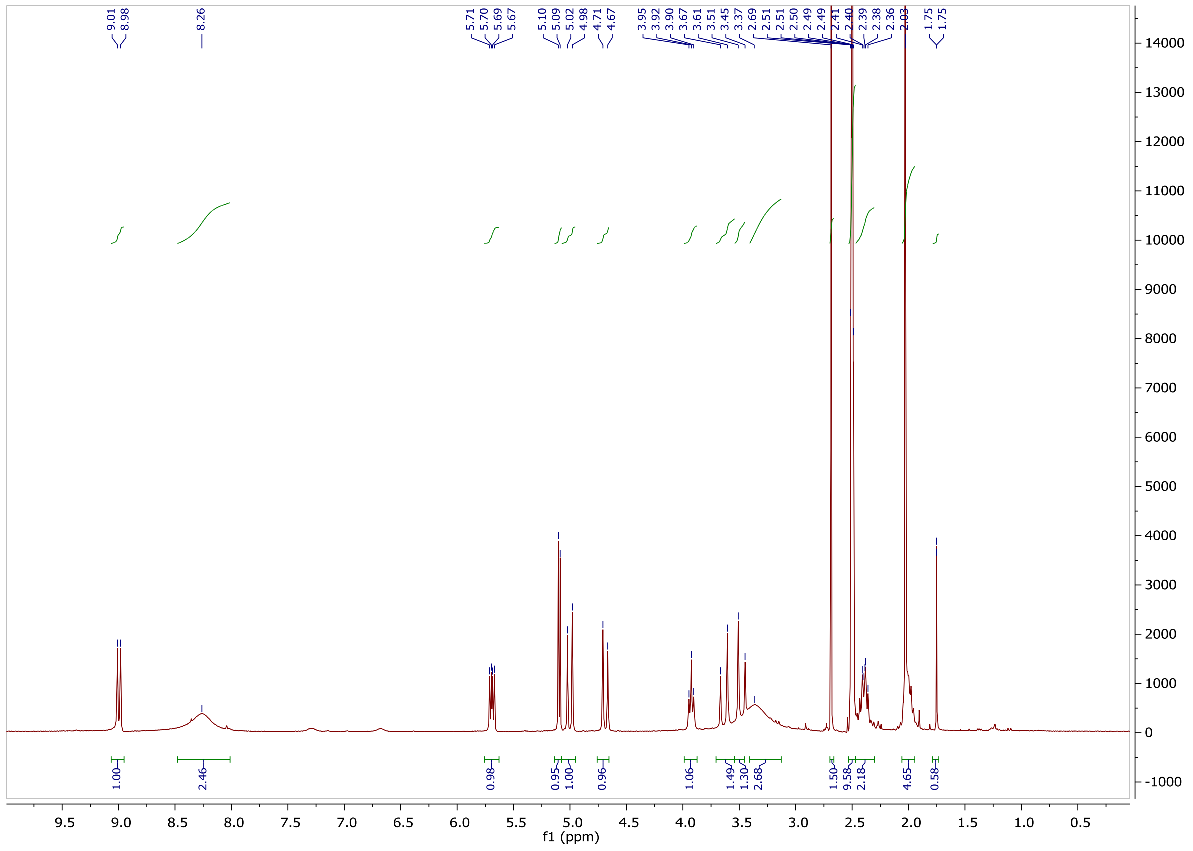

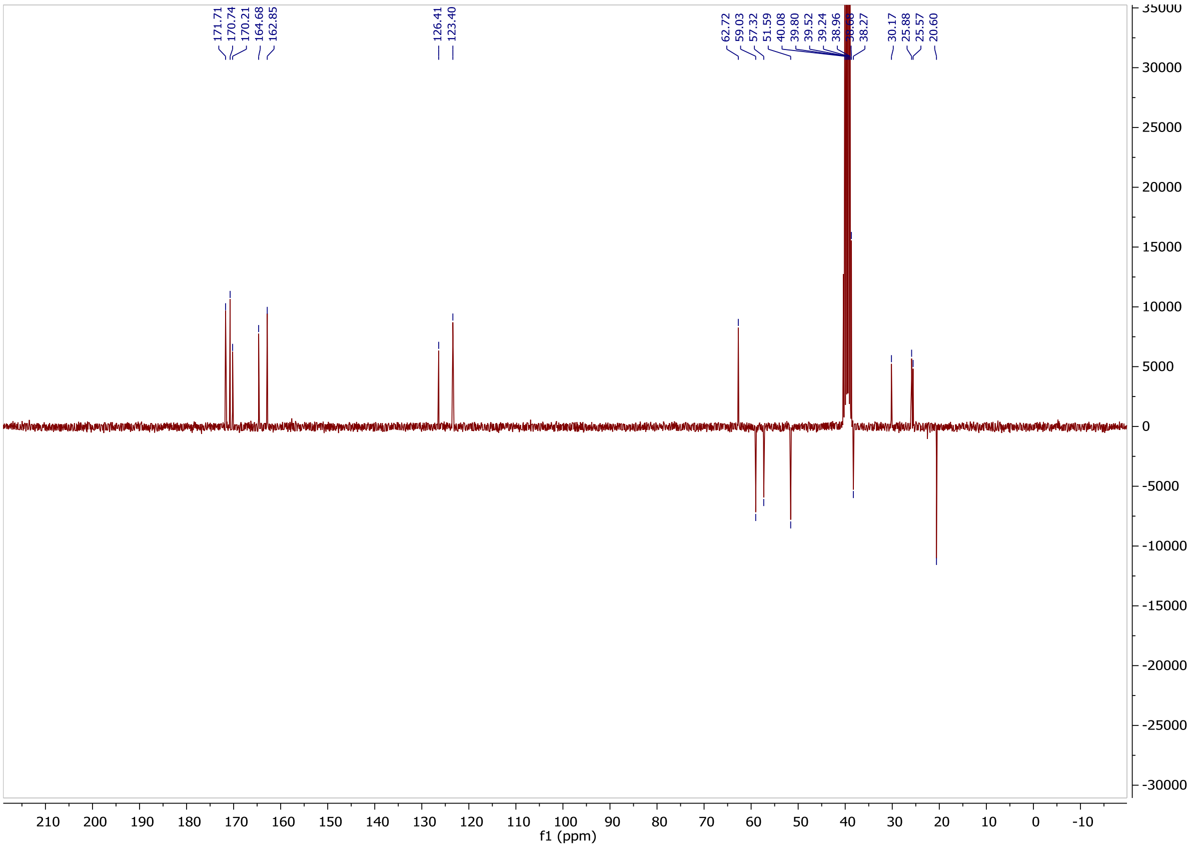

7-aminocephalosporanic acid – aminovaleric acid (4)

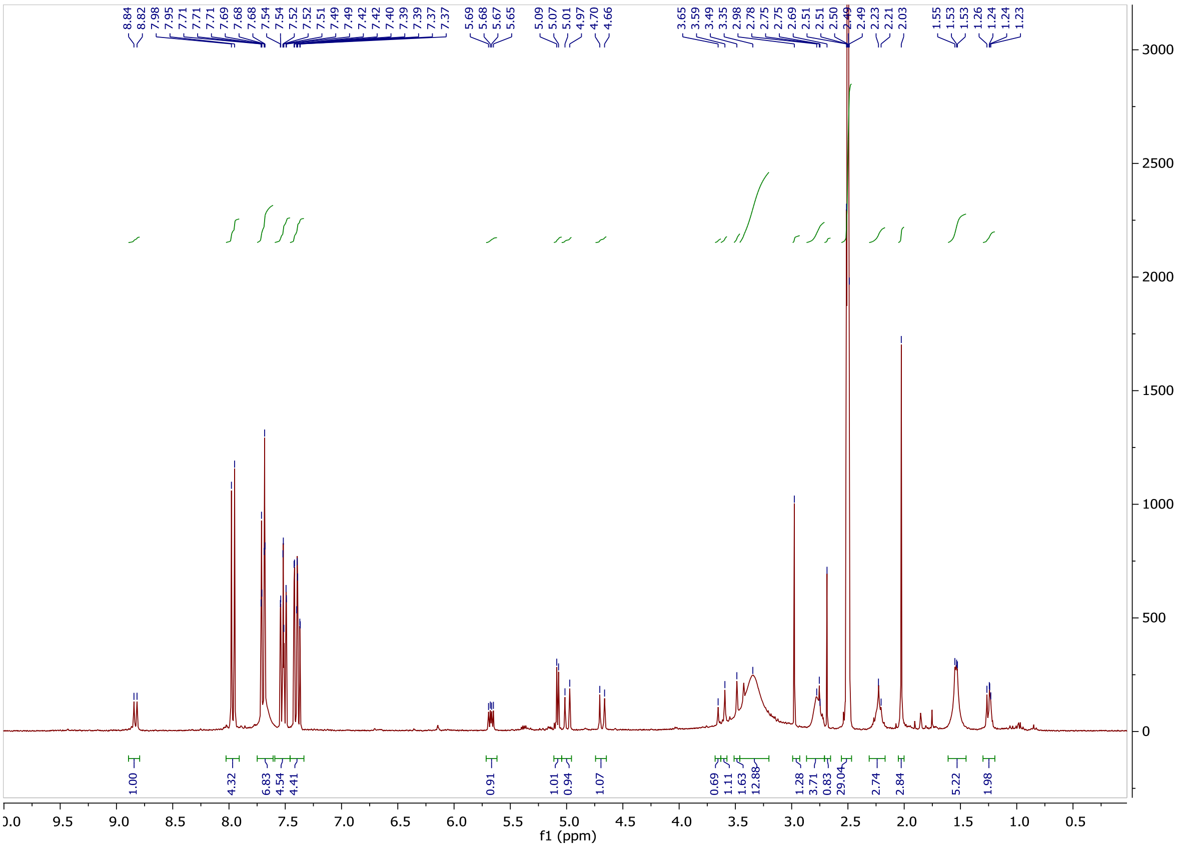

References

1 Kim, L. *et al.* Structural and Biochemical Study of the Mono-ADP-Ribosyltransferase Domain of SdeA, a Ubiquitylating/Deubiquitylating Enzyme from Legionella pneumophila. *Journal of Molecular Biology* **430**, 2843-2856 (2018). https://doi.org:10.1016/j.jmb.2018.05.043

2 El Oualid, F. *et al.* Chemical Synthesis of Ubiquitin, Ubiquitin-Based Probes, and Diubiquitin. *Angewandte Chemie* **122**, 10347-10351 (2010). https://doi.org:10.1002/ange.201005995

3 Barrio, J. R., Secrist, J. A. & Leonard, N. J. A Fluorescent Analog of Nicotinamide Adenine Dinucleotide (fluorescent coenzyme/quenching/enzyme activity/NAD +/1,N6-ethenoadenosine derivative). 2039-2042 (1972).

4 Liu, R. *et al.* A Synthetic Dual Drug Sideromycin Induces Gram-Negative Bacteria to Commit Suicide with a Gram-Positive Antibiotic. *Journal of Medicinal Chemistry* **61**, 3845-3854 (2018). https://doi.org/10.1021/acs.jmedchem.8b00218
